# The Micro-Immunotherapy Medicine 2LEID Exhibits an Immunostimulant Effect by Boosting Both Innate and Adaptive Immune Responses

**DOI:** 10.3390/ijms23010110

**Published:** 2021-12-22

**Authors:** Camille Jacques, Mathias Chatelais, Karim Fekir, Louis Fauconnier, Manon Mellier, Dieudonnée Togbe, Ilaria Floris

**Affiliations:** 1 Preclinical Research Department, Labo’Life France, 1 Rue François Bruneau, 44000 Nantes, France; ilaria.floris@labolife.com; 2 ProfileHIT SASU, 7 Rue du Buisson, 44680 Sainte-Pazanne, France; mathias.chatelais@profile-hit.com (M.C.); karim.fekir@profile-hit.com (K.F.); 3 ArtImmune SAS, 13 Avenue Buffon, 45100 Orleans, France; louis.fauconnier@artimmune.com (L.F.); manon.mellier@artimmune.com (M.M.); 4 Centre National de la Recherche Scientifique (CNRS), Unité Mixte de Recherche 7355 (UMR7355), 3 B Rue de la Ferollerie, CEDEX 2, 45071 Orleans, France; dieudonnee.togbe@cnrs-orleans.fr; 5 Experimental and Molecular Immunology and Neurogenetics, University of Orleans, Rue de la Ferollerie, CEDEX 2, 45100 Orleans, France

**Keywords:** low dose, micro-immunotherapy, 2LEID, immunostimulant drug, respiratory infectious diseases, *influenza A* virus, host defenses, innate and adaptative immunity

## Abstract

This study aimed at evaluating the effects of the micro-immunotherapy medicine (MIM) 2LEID, both in vitro and in vivo, on several components of the innate and adaptive immune system. MIM increased the phagocytic activity of macrophages, and it augmented the expression of the activation markers CD69 and HLA-DR in NK cells and monocytes/macrophages, respectively. The effect of MIM was evaluated in a model of respiratory infection induced by *influenza A* virus administration to immunocompetent mice in which it was able to improve neutrophil recruitment within the lungs (*p* = 0.1051) and slightly increased the circulating levels of IgM (*p* = 0.1655). Furthermore, MIM stimulated the proliferation of CD3-primed T lymphocytes and decreased the secretion of the immunosuppressive cytokine IL-10 in CD14^+^-derived macrophages. Human umbilical vein endothelial cells were finally used to explore the effect of MIM on endothelial cells, in which it slightly increased the expression of immune-related markers such as HLA-I, CD137L, GITRL, PD-L1 and ICAM-1. In conclusion, the present study suggests that MIM might be a promising nonspecific (without antigen specificity) immunostimulant drug in preventing and early treating respiratory infections, but not only exclusively, as it would gently support several facets of the immune system and host defenses.

## 1. Introduction

Respiratory tract infections are a heterogeneous group of conditions affecting the upper and/or lower respiratory tract and can be caused by micro-organisms or pathogen agents such as bacteria (*Streptococcus pyogenes*) or viruses (*influenza A*, *coronaviruses*, *rhinoviruses*, *adenoviruses*, *enteroviruses*). Respiratory infectious diseases can affect the sinuses, throat, airways or lungs. In particular, those that affect the lower respiratory tract tend to be more serious than those that stay confined to the upper respiratory tract [1]. Infections of the respiratory system encompass diverse illnesses including the common cold, acute bronchitis, influenza or respiratory distress syndromes, the commonly associated symptoms comprising runny/plugged nose, sneezing, sore throat, cough, hoarseness, facial pressure and low-grade fever. As infection from one individual to another occurs through the inhalation of contaminated droplets, the first line of defense towards those conditions remains prevention measures, such as hand washing, superficies sanitization, wound disinfection or, in the case of definite symptoms, self-isolation or the use of disposable tissues. Unless they are from bacterial origin, or if bacterial complication occurs, most respiratory infections are self-limited, so antibiotics are useless and no evidence-based data support their effectiveness in alleviating the manifestations of the common cold in children and adults [2,3]. So far, current treatments mainly aim at symptom relief, and over-the-counter topical/oral nasal decongestants, alone or in combination with antihistamines, cough suppressants and expectorants, can be used to help the body fight against those symptoms [4]. A plethora of factors related to modern life, such as chronic stress, pollution, poor food choices, nutrient deficiencies or sleep deprivation, can progressively alter the immune system and the host defenses, thus facilitating the spreading of the pathogens within the organism [5,6,7,8]. To date, no standardized treatment aiming at their prevention and acting as immunostimulant exists, mostly due to the heterogeneity of these diseases’ etiology. However, it has been shown that, taken as a preventive medication, daily oral doses of 0.2 g or more of vitamin C reduced the duration of the common cold by 8% in adults and by 13% in children, as well as reducing its severity [9]. Moreover, according to an American survey, prophylaxis motives such as “prevention of cold/influenza” and “immune boosting” ranked amongst the top eight reasons to take vitamin/minerals and herbal supplements [10].

In this context, micro-immunotherapy (MI), which is a therapeutic approach aiming at gently modulating the body’s homeostasis by maintaining and/or restoring immune system functions [11,12,13,14], could be a great asset in the management of respiratory infectious diseases. All the MI medicines’ formulations combine different types of active ingredients that can be classified according to their roles and characteristics as follows: (1) chemical mediators directly involved in immune responses, such as cytokines; (2) other signaling molecules playing important roles in cell–cell communication under physiological and pathological conditions, such as growth factors, hormones and neuropeptides; and (3) nucleic acids, either under their original state (total DNA and RNA, extracted from *Pinus halepensis* and *Foeniculum vulgare*, respectively) or under the form of specific nucleic acids (SNA) [11,12,13,14,15,16]. The SNA therapeutic innovation developed by Labo’Life and protected by the European Patent EP0670164B1 consists of single-stranded DNA molecules (16–44 bases) specifically designed to target one or more gene and/or transcript sequences, according to paired-base complementarity. The active ingredients employed in MI medicines are prepared at low doses (LDs) or ultralow doses (ULDs) with the aim of modulating specific proteins involved in the onset and/or the development of a pathology/clinical condition by inducing either an upregulation or a downregulation in these proteins, with the aim of reestablishing their homeostatic levels in the organism [16].

MI medicines are sucrose–lactose pilules (also called globules) for oromucosal administration, enclosed into capsules, having the composition expressed as number of centesimal Hahnemannian dilution (CH) like other homeopathic medicinal products. The pharmaceutical manufacturing method complies with the European Pharmacopeia monographs 1038 and 2371, current edition. It consists of repetition series of a two-step process: (1) a 1/100 dilution followed by (2) a calibrated vertical shaking. This procedure, which is called “sequential kinetic process” (SKP), is reproduced until reaching the desired number of CH for each active substance (see Supplementary Figure S1: a scheme to explain the manufacturing process, including the SKP, is provided).

2LEID (referred to as MIM in the paper), the medication investigated in this preclinical study, is available on the market in Belgium, Spain and Italy. MIM consists of a sequence of 10 capsules. Each of the 10 capsules envelops about 380 mg of sucrose–lactose pilules and owns its unique combination of the active ingredients. The overall MIM composition is described in Table 1 and in the following text: (1) human recombinant (hr) cytokines and growth factors, namely hr-interleukin (IL)-1β (5 or 10 CH), hr-IL-2 (5 or 10 CH), hr-IL-5 (6 or 10 CH), hr-IL-6 (6 or 10 CH), hr-interferon (IFN)-γ (6 or 10 CH), transforming growth factor (TGF)-β (10 or 30 CH) and tumor necrosis factor (TNF)-α (5 or 10 CH), and (2) nucleic acid based-molecules (RNA and DNA at 8 or 10 CH) and SNA-HLA I, SNA-HLA II and SNA-EID at 10 or 16 CH (designed to target human leukocyte antigen (HLA)-I, HLA-II and IL-2, respectively).

MIM, like all the other MI medicines, is intended to be taken through oromucosal administration, in a fasted state, to optimize the delivery of the active substances to the lymphoid tissues of the buccal mucosae. The oral cavity possesses its proper local immune system, where the residing immune cells, such as dendritic cells and antigen-presenting cells, can initiate and orchestrate immune responses [17]. In particular, as reported by A.-H. Hovav, the human oral mucosae is characterized by several dendritic cell subsets, such as Langerhans cells, myeloid dendritic cells and plasmacytoid dendritic cells, each displaying their own phenotypes and engaging their unique mechanisms for immune induction and modulation [18]. Regarding the pivotal role of these cells, MIM may mediate their biological effects by directly interacting with those particular cells as well. Moreover, there is evidence supporting the fact that oral intake of cytokines and growth factors is an effective way to modulate the body’s natural defenses. In particular, as the specific formulation of MIM includes IL-1β, IFN-γ and TNF-α, which were all shown to enhance the phagocytosis capabilities of neutrophils, our interest focused first on the innate side of the immunity (Table 1, middle column) [19]. Moreover, IFN-γ and TNF-α cooperate and have synergistic actions in activating the immune system, including antiviral defenses, for example by stimulating macrophage functions [20,21]. Cooperative activation of proinflammatory agents profoundly influences the immune response to infections, as well as the efficiency of cellular clearance mechanisms involved in tissue repair and resolution of inflammation. For this reason, the capsule of MIM containing IFN-γ and TNF-α at 6 and 5 CH (Table 1, right column) has been tested in vitro to assess its capacity to stimulate innate immune responses (phagocytosis capabilities, cell surface markers and cytokine secretion levels). In the context of infections caused by viruses, activated macrophages and natural killer (NK) cells, in turn, produce IFN-γ, contributing to Th1 cell differentiation in activating adaptive immune responses [22,23].

First, the in vitro capability of MIM to stimulate *Candida albicans* (*C. albicans*) phagocytosis was evaluated in human macrophages, and the effects of MIM on CD14^+^-derived macrophage viability, surface markers and cytokine secretion were investigated. Furthermore, MIM was evaluated in an in vivo preclinical model of respiratory infectious disease induced by *influenza A* virus (IAV) administration to immunocompetent BALB/cJRj mice. Human primary peripheral blood mononuclear cells (PBMCs) were used to appraise MIM’s ability to stimulate both innate and adaptive immune cells. Their proliferation and activation were analyzed by flow cytometry. The same technique was used to evaluate the impact of MIM on the expression profile of immune-related membrane markers in endothelial cells. Overall, the main objective of this study was to investigate the effects of the MIM on several immune system components involved in the organism’s defense (innate and adaptive immune responses) against the *influenza A* virus by using in vitro and in vivo models.

## 2. Results

### 2.1. MIM Stimulates the Phagocytosis Capabilities of Macrophages In Vitro

**Figure 1:**
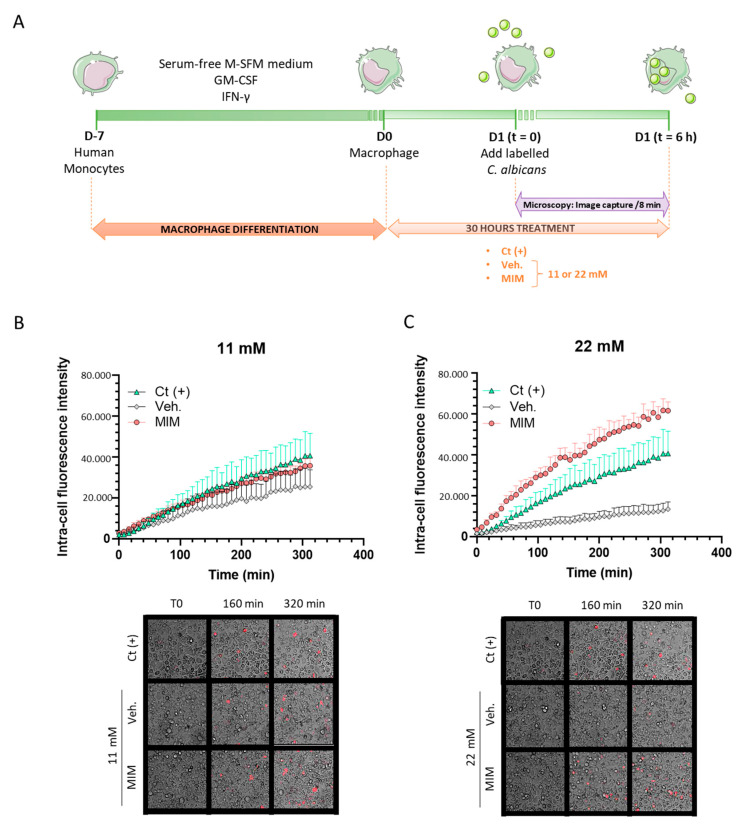
MIM effects on the phagocytosis capabilities of monocyte-derived macrophages. (**A**) Schematic representation of the experimental protocol. (**B**,**C**) Upper panels: Monocytes isolated from one healthy donor were differentiated into macrophages for 6 days in presence of GM-CSF and IFN-γ. The treatment with MIM or vehicle at either (**B**) 11 mM or (**C**) 22 mM started 24 h before inducing the phagocytosis. Each condition was performed in 6 replicates. Data are represented as mean ± SD. (**B**,**C**) Lower panels: Representative pictures of the wells at different time points. Macrophages are visualized in bright light and phagocytosed *C. albicans* correspond to the red spots.

As one hallmark of a healthy immune system is its defense capacity against pathogens through phagocytosis, we first wanted to focus our analysis on assessing the effects of MIM on this innate-related part of the immune reaction. Human monocytes isolated from one healthy donor were cultivated for seven days in a macrophage-differentiating medium and treated for 24 h with MIM or the vehicle at either 11 or 22 mM, prior to adding heat-killed—pHRodo Red-labeled—*C. albicans* for 30 min (Figure 1A). As the pHRodo Red dye only emits fluorescence in an acidic milieu, any increase in the signal intensity evidences an increased phagocytosis activity as the dye penetrates the phagosome. Fluorescence was monitored every 8 min over a 6 h period after the initial 30 min incubation of the labeled yeast, and the results are presented in Figure 1B,C as the mean ± SD of six replicates per condition. As the phagocytosis rate slightly increased over time for both of the tested concentrations in basal vehicle-treated conditions (grey dots), MIM (red dots) led to a higher level of phagocytosis than the control conditions. After 312 min, the phagocytosis capabilities were indeed 1.4 and almost 4.6 times higher than the vehicle-treated macrophages at 11 and 22 mM, respectively. Of note, at 22 mM, the intensity of the signal was even stronger than the one induced by the “Ct (+)” phagocytosis-inducer positive control (green dots). Being performed once, no statistical inference was done, as mentioned in Section 4. Even if more investigations are needed to confirm the statistical significance of these findings, these results still suggest that MIM displays a stimulatory effect on the macrophages’ ability to phagocytose pathogens.

### 2.2. MIM Reduces the Expression of Cell Surface Markers on CD14^+^-Derived Macrophages and the Secretion of Anti-Inflammatory Cytokines without Affecting Cell Viability

**Figure 2:**
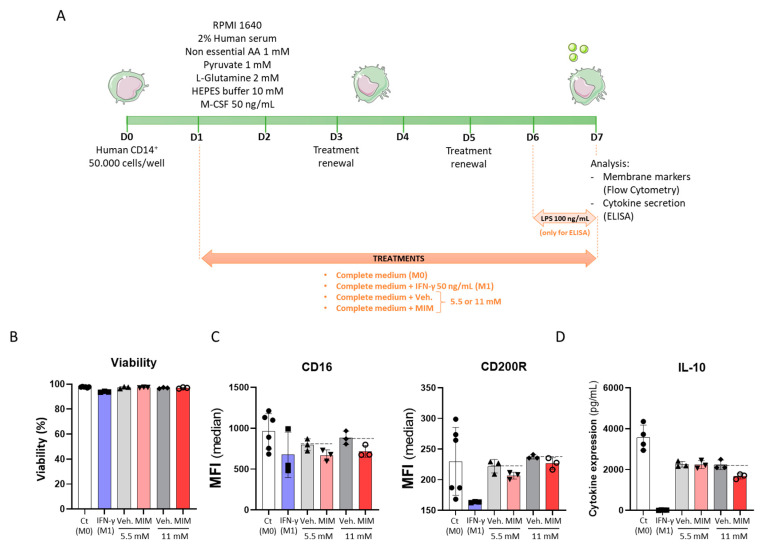
MIM modulates CD16 and CD200R expression and IL-10 secretion of CD14^+^-derived macrophages in vitro without affecting the cell viability. (**A**) Representative scheme of the experimental protocol. CD14^+^ cells were obtained from PBMCs isolated from one healthy donor and cultivated for 6 days in complete medium supplemented with M-CSF 50 ng/mL (M0) and in presence of either MIM or vehicle at 5.5 or 11 mM. CD14^+^ cells cultivated in complete medium in which M-CSF 50 ng/mL and IFN-γ 50 ng/mL were added served as a positive control towards M1 macrophage-differentiation (M1). A 24 h LPS treatment (100 ng/mL) was applied for the ELISA test as an inducer of an inflammatory status. Viability and cell surface marker expression were assessed by flow cytometry and IL-10 concentration was evaluated by ELISA. AA: amino acids. (**B**) Effects of MIM on cell viability. The cell viability was assessed by flow cytometry, as the NIR-Zombie positive cells. (**C**) Effects of MIM on the expression of the cell surface markers CD16 and CD200R and (**D**) the secretion of IL-10. Data are represented as mean ± SD.

Regarding the fact that MIM exerted stimulatory effects on the biological function of macrophages after a short incubation period of only 24 h, we then evaluated whether MIM could also act in supporting macrophage polarization, which is an in vitro process modeled by longer incubation periods [24]. Evidence showed that macrophages are key players in the progression of respiratory tract infections, and their polarization towards an M1 phenotype is characterized by high levels of proinflammatory cytokines [25,26]. In our study, freshly isolated human CD14^+^ cells were thus cultured in basal M0 conditions (complete medium + M-CSF 50 ng/mL), with MIM/Veh. at either 5.5 or 11 mM for 6 days (see scheme in Figure 2A). The cell viability and the expression levels of CD16 and CD200R were then assessed by flow cytometry. Cells cultivated in the same basal M0 medium supplemented with recombinant IFN-γ at 50 ng/mL were used as M1 macrophage-differentiation control conditions. As illustrated in Figure 2B, MIM did not present any toxicity on the CD14^+^-derived macrophages, as it did not affect the cell viability after 6 days in culture. Regarding the expression of cell markers (Figure 2C), our results showed that MIM tends to slightly decrease the expression of both CD16 and CD200R after 6 days of treatment at either 5.5 or 11 mM, compared with the sucrose–lactose pills alone. Decreases of 16.2% and 18.7% in the expression of CD16 were noticed at the concentrations of 5.5 and 11 mM, respectively, whereas decreases of 6.9% and 4.5% in the expression of CD200R were observed at those same concentrations. The trend observed in these conditions was similar to the effect of IFN-γ 50 ng/mL used as an M1 control, which also led to a reduction in CD16 and CD200R levels, but with a higher magnitude (by 30% and 28.8%, respectively). In order to induce a proinflammatory stimulus, CD14^+^-derived macrophages were cultured in the presence of 100 ng/mL lipopolysaccharide (LPS). As illustrated in Figure 2D, MIM reduced the secretion of the anti-inflammatory cytokine IL-10 (by 25.1% at the concentration of 11 mM, when compared with the vehicle), while it did not show any effect at 5.5 mM. IFN-γ-treated CD14^+^-derived macrophages also displayed a reduction in IL-10 secretion (by 99.6%) compared with the M0 control. Finally, the medicine was not able to induce any upregulation of TNF-α or IFN-γ in our CD14^+^ cells (data not shown). The experiment was performed once; thus, more investigations are needed to confirm the statistical significance of the findings. However, taken together, these results showed that the expression of CD16 and CD200R and the secretion of the anti-inflammatory cytokine IL-10 were downregulated by MIM, in the same manner as the IFN-γ-mediated M1-polarized macrophages used as control. Even if such results definitely do not allow the conclusion that MIM acts as an M1 macrophage polarization inducer, they tend to highlight the potential of MIM to modulate macrophage phenotype, somewhat reflecting their possible engagement towards an M1-differentiated macrophage profile.

### 2.3. Preventive Treatment MIM Improves Antibody Response at Both Local and Systemic Levels in Influenza A Virus-Induced Respiratory Infection

**Figure 3:**
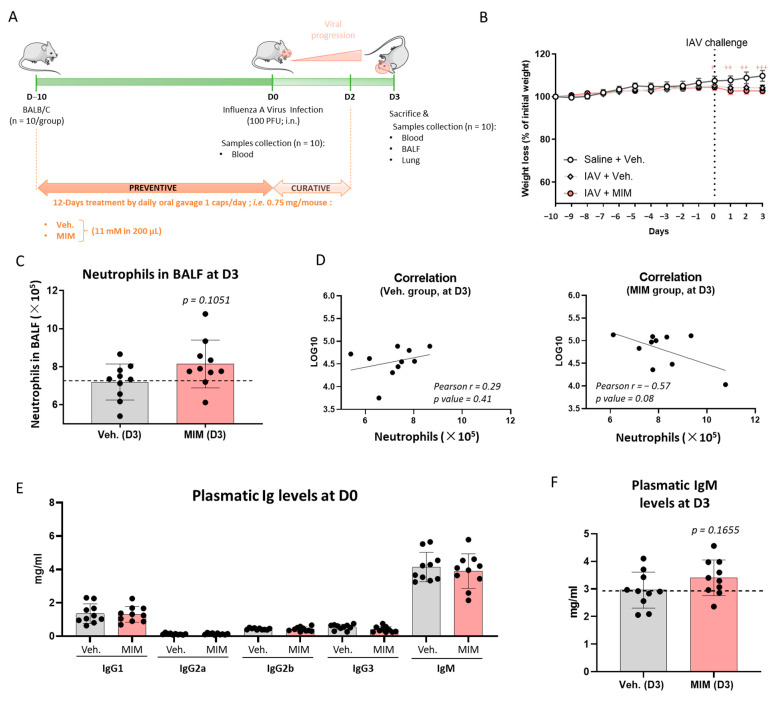
MIM can help the recruitment of neutrophils to the lungs and increases the plasmatic levels of IgM in an in vivo model of respiratory infection induced by *influenza A* virus (IAV). (**A**) Representative scheme of the general experimental protocol. MIM or vehicle (Veh.) at 11 mM was administrated daily by oral gavage from Day–10 (D–10) to Day 2 (D2) to BALB/cJRj female mice (n = 10 mice per group). The animals were intranasally (i.n.) infected with IAV (100 PFU) on D0, one hour after MIM or Veh. administration. Mice were euthanized on Day 3 (D3) and blood, bronchoalveolar fluid (BALF) and lungs were collected. (**B**) Effect of MIM treatment on body weight changes has been investigated. Data are mean ± SEM of 10 mice per group. Statistical evaluation of differences between the experimental groups was determined by using two-way ANOVA followed by a Dunnett’s multiple comparisons post-test. *p* values of less than 0.05 were considered significant. + *p* < 0.0332, ++ *p* < 0.0021, +++ *p* < 0.0002. The statistical analysis was performed with GraphPad Prism, Version 9 for Windows (GraphPad Software Inc., San Diego, CA, USA, www.graphpad.com accessed on 29 July 2021). (**C**) Neutrophil recruitment in BALF on D3. Neutrophil counts in BALF were analyzed and the represented data are the mean ± SD of 10 mice per group. Nonparametric Mann–Whitney test was performed (*p* = 0.1051) to compare both groups. (**D**) Correlations between the viral load in the lungs and the number of neutrophils in the BALF on D3 in the groups treated with vehicle (left panel) and MIM (right panel). Pearson’s r scores and *p* values are depicted in each graph. (**E**) Plasmatic immunoglobulin levels of IgG1, IgG2a, IgG2b, IgG3 and IgM on D0. (**F**) Plasmatic immunoglobulin levels of IgM on D3 post-IAV. IgM was analyzed and the represented data are the mean ± SD of 10 mice per group. Nonparametric Mann–Whitney test was performed (*p* = 0.1655) to compare both groups.

The tested MIM is used to boost the immune system to prevent bacterial and viral infections. As it is prescribed as a preventive and curative treatment for respiratory infections including IAV threat, we sought to model such conditions in vivo by administrating immunocompetent BALB/cJRj mice with either MIM or the vehicle sucrose–lactose pilules at 11 mM (0.75 mg/mouse), by daily oral gavage, 10 days prior to intranasal inoculation of IAV and up to 2 days after the viral challenge. The entire protocol lasted 13 days, as depicted in Figure 3A. Animals’ body weight was monitored during the course of the whole experiment (Figure 3B). Even though the uninfected control group (Saline + Veh.) kept increasing in body weight by about 5% from Day 0 (D0) to Day 3 (D3), the animals challenged with the virus displayed a weight stabilization from D0 to D3, and no difference in weight was observed between the vehicle and the MIM groups. In order to assess if the treatment had an effect at a local level, within the respiratory tract, and impacted the inflammatory immune cell recruitment to the bronchoalveolar tract, bronchoalveolar lavage fluid (BALF) was collected on D3 post-IAV and the different immune cell populations were counted (i.e., total number of cells, lymphocytes, neutrophils, eosinophils and macrophages). None of the analyzed immune cell populations displayed any change between groups (data not shown). Regarding the neutrophil population, a slight increase in number was noticed in the group treated with MIM compared with the control one, with a nonsignificant *p* value of 0.1051 (Figure 3C). The measure of the viral load within the lungs was also assessed by qPCR on D3 post-IAV and no significant change was observed between the groups (data not shown). However, a negative close to significant correlation (Pearson’s r = −0.57; *p* value = 0.08) was found between the viral load in the lungs and the number of neutrophils in the BALF in the MIM-treated group only (Figure 3D).

To investigate if MIM exhibited systemic effects on the humoral response, the levels of circulating immunoglobulins (Igs) at D0 and D3 post-IAV were also assessed. MIM used as a preventive treatment did not exert any effect on the humoral response as no difference in the circulating levels of IgG1, IgG2a, IgG2b, IgG3 and IgM was found between treated and untreated groups on D0 (Figure 3E). Interestingly, on D3, a close to significant (*p* value = 0.1655) increase in the plasmatic IgM levels was noticed in the MIM group compared with the vehicle group (Figure 3F). Apart from IgM, none of the other analyzed Igs showed variations in their circulating levels (data not shown). Again, the in vivo experiment was performed once. Indeed, more investigations are still needed to confirm the statistical significance of these findings. However, as this model allows mimicking the physiological immune response to a virus operating in upper airways, the results, even if nonsignificant, suggest that MIM may have a local effect on neutrophil recruitment in the BALF, as well as a systemic effect in increasing the levels of circulating IgM, thus revealing its possible beneficial effects as a preventive treatment on both sides of the innate and adaptive immunity.

### 2.4. MIM Increases the Proliferation and the Activation of PBMCs In Vitro

**Figure 4:**
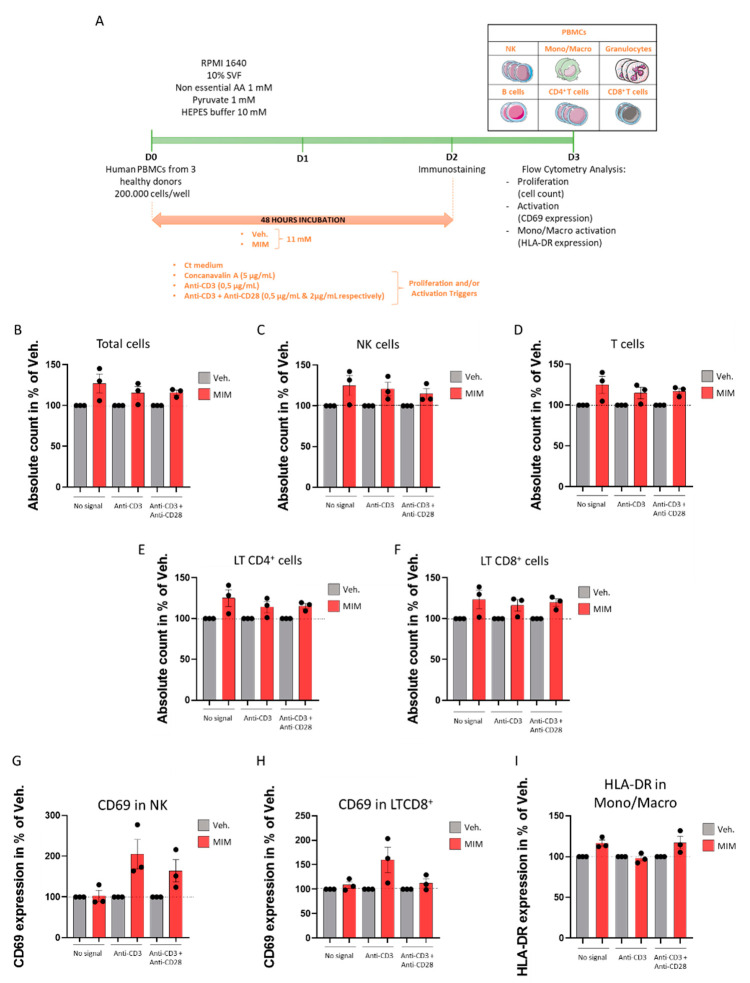
MIM increases the proliferation and the activation of PBMCs in vitro. (**A**) Representative scheme of the experimental protocol. Human PBMCs from three healthy donors were cultivated for 48 h in (i) classical culture condition (Ct medium, referred to as “no signal”), (ii) classical culture conditions plus anti-CD3 antibody at 0.5 µg/mL (referred to as “anti-CD3”) or (iii) classical culture conditions plus anti-CD3 antibody at 0.5 µg/mL + anti-CD28 antibody at 2 µg/mL (referred to as “anti-CD3 + anti-CD28”) in presence of MIM/Veh. at 11 mM. The cells were immune-stained on D2 and analyzed by flow cytometry on D3. The total number of cells and the total cell count within each subpopulation (NK, monocytes/macrophages, granulocytes, B cells, T cells, CD4^+^ T cells, CD8^+^ T cells) and their activation status were assessed. Each cell subpopulation was discriminated according to the expression of the markers given Section 4. The expression of the CD69 marker was evaluated in each subpopulation as the activation marker of reference. The expression of HLA-DR was assessed within the monocytes/macrophages subpopulation as a supplementary activation marker. AA: amino acids. The total number of cells (**B**), the NK cell count (**C**), the T cell count (**D**), the CD4^+^ T cell count (**E**), and the CD8^+^ T cell count (**F**) were evaluated in the conditions defined in (i), (ii) and (iii). Grey-shaded histograms represent the vehicle (Veh.) and red-shaded histograms represent MIM treatment at 11 mM. Each histogram represents the mean ± SEM of the cell count obtained for each individual donor, as a percentage of the MIM-condition count normalized to the Veh. condition. Each individual point (black dot) represents the average of a triplicate per donor. (**G**) MIM induces the expression of CD69 in NK cells and in CD8^+^ T cells (**H**). Each histogram represents the mean ± SEM of the CD69 expression obtained for each individual donor, as a percentage of the MIM-condition CD69 expression normalized to the Veh. condition. Each individual point (black dot) represents the average of a triplicate per donor. (**I**) MIM induces the expression of HLA-DR in monocytes/macrophages. Each histogram represents the mean ± SEM of the HLA-DR expression obtained for each individual donor, as a percentage of the MIM-condition HLA-DR expression normalized to the Veh. condition. Each individual point (black dot) represents the average of a triplicate per donor.

As the tested MIM contains in its formulation key regulators involved in immune cell viability, proliferation and functions, we wanted to address its capabilities to stimulate the proliferation and the activation of human PBMCs in both contexts: in a “naïve” basal state (i.e., preventive effect of the MIM) and in a preprimed immune state in which T cells would already be activated (i.e., therapeutic supportive effect of the MIM). T cell activation is subjected to distinct signals, the first one being the presentation of peptide–antigen to the antigen receptor complex (TCR), together with additional costimulatory signals from coreceptors, such as CD28 [27]. Thus, a model of activating antibodies to CD3 and CD28, used as antigen-independent signaling for the TCR complex and costimulation trigger, respectively, was employed to mimic the preprimed immune state [28]. As illustrated in Figure 4A, human PBMCs freshly isolated from three healthy donors were thus cultivated in presence of 11 mM MIM/Veh. for 48 h in (i) classical culture conditions (referred to as “no signal” in the graphs) or in preprimed conditions modeled by either (ii) the presence of the anti-CD3 antibody alone (referred to as “anti-CD3”) or (iii) the presence of both the anti-CD3 and anti-CD28 antibodies (referred to as “anti-CD3 + anti-CD28”). The PBMCs were also cultivated in classical culture conditions in presence of concanavalin A as an additional internal proliferation control (Supplementary Figure S2). Total cells and the different immune cell subpopulations depicted in the cartoon in Figure 4A (NK cells, monocytes/macrophages, granulocytes, B lymphocytes (B cells), T lymphocytes (T cells), CD4^+^ T cells and CD8^+^ T cells) were counted by flow cytometry and their activation status was monitored through their CD69 expression levels. The results presented in Figure 4B show that, in basal conditions (“no signal”) and in the two preprimed conditions tested (“anti-CD3” and “anti-CD3 + anti-CD28”), MIM was able to induce the proliferation of the total PBMCs. Moreover, MIM acted in stimulating the proliferation of the NK cells (Figure 4C) and the T cell populations of both CD4^+^ and CD8^+^ T lymphocytes (Figure 4D–F). However, it is important to state that the experiment was performed once for each donor; indeed, more investigations are still needed to confirm the statistical significance of these findings. It is finally also worth mentioning that regarding the other PBMC subpopulations, MIM displayed a slight proliferative-enhancing effect on monocytes/macrophages, granulocytes and B cells in basal conditions only (data not shown). Data of the absolute count obtained for each individual donor are presented in Supplementary Figure S2. Regarding the effect of MIM on the activation status of the PBMCs, even if MIM did not show any effect on the CD69 expression in basal conditions, it enhanced the CD69 expression of the NK cells in the two preprimed conditions assessed (Figure 4G), and it also enhanced the CD69 expression in the CD8^+^ cells, but only in the presence of the anti-CD3 alone (Figure 4H). Data of the CD69 expression obtained for each individual donor are presented in Supplementary Figure S3. Finally, as the results obtained in the previous sections of this study emphasized the MIM’s effects on the monocytes/macrophages (Figure 1 and Figure 2), and considering the fact that HLA-DR expression is reduced on monocytes during severe respiratory syncytial virus infections [29], we also wanted to assess the MIM effect on the expression of HLA-DR in this subpopulation. The results presented in Figure 4I and Figure S4 consistently demonstrated that MIM tends to slightly increase HLA-DR expression in the population of monocytes/macrophages in basal conditions and in presence of the cocktail of the two stimulatory antibodies. Even if the trends found here in three healthy donors need to be statistically confirmed, these overall results highlight that MIM increases the proliferation of PBMCs independently of a preexisting background of primed immune cells. Additionally, it seems that MIM would also enhance the expression of activation markers on NK cells, CD8^+^ T cells and monocytes/macrophages, the magnitude of its effects depending on the presence of an initial stimulating factor, which suggests that MIM could play the role of a costimulatory signal for these cells.

### 2.5. MIM Modulates the Expression of Endothelial Cell Surface Markers In Vitro

**Figure 5:**
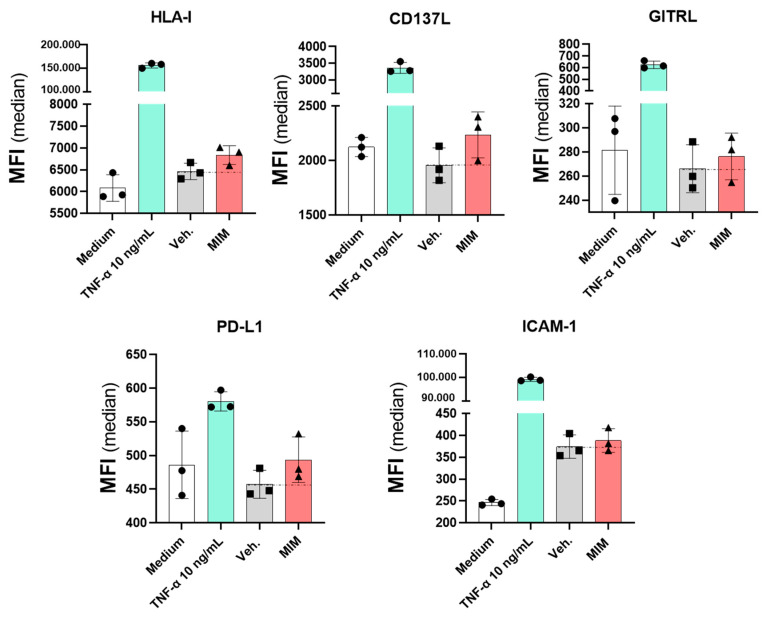
MIM modulates the expression of immunity-related endothelial cell surface markers. MIM modulates the expression levels, expressed as MFI, of several endothelial cell surface markers (HLA-I, CD137L, GITRL, PD-L1 and ICAM-1). HUVECs were incubated for 48 h in presence of either MIM or vehicle (Veh.) at 11 mM. TNF-α at 10 ng/mL was used as a positive control. The results represent the mean ± SD of one technical triplicate for each condition.

Besides their role in angiogenesis, coagulation and vascular homeostasis, endothelial cells constitute a key component of the immune response, as they express a range of immune-related markers and molecules that allow them to respond to inflammatory stimulations [30]. Their functions as vascular permeability regulators also actively implicate them in the recruitment and the extravasation of the immune mediators at the site of infection, facilitating the body’s defense mechanisms against aggressions. Performing a profiling of endothelial cell membrane markers can thus give valuable information about how MIM can impact the interplay between the immune cells, even before the onset of a respiratory infection, in the context of preventive therapy with this medicine. A human endothelial cell model, HUVECs, was thus chosen to decipher to what extent MIM affects the expression of immunity-related membrane markers, which may help in explaining the effects of this medicine previously observed in vivo at the systemic level (Figure 3). In an attempt to choose a concentration of sucrose–lactose that does not impact the cell viability and could interfere with the profiling experiment, HUVECs were first treated for 48 h with increasing concentrations (ranging from 0.34 to 11 mM) of either the sucrose–lactose vehicle pill alone or the MIM-impregnated one. Cell viability was assessed by flow cytometry (data not shown). As the highest dose tested (11 mM) did not impact the viability, it was chosen for the further profiling analysis. As described in Section 4, HUVECs were then treated for 48 h with 11 mM of either MIM or the vehicle alone, the media and the corresponding treatments being renewed once, after the first 24 h incubation period. The expression of five cell surface markers (HLA-I, CD137L, GITRL, PD-L1 and ICAM-1) was analyzed by flow cytometry. Tumor necrosis factor-α at 10 ng/mL was used as a positive control, as studies have shown its role as a proper inducer of the expression of these markers [31,32,33,34]. The expression of the five analyzed markers was slightly increased by MIM compared with the vehicle-treated cells (Figure 5). More precisely, the expression of HLA-I was increased by 5.8%, CD137L by 14.3%, GITRL by 3.8%, PD-L1 by 7.9% and ICAM-1 by 3.7%, compared with the vehicle alone. This last experiment was performed once, and more investigations are still needed to confirm the statistical significance of these findings. Nevertheless, regarding the fact that the endothelial cells are at the cornerstone between the blood circulation and the immune system, such modulations are another indicator of the possible crosstalk between those cells and the PBMCs and may partially explain the systemic effects of MIM observed in vivo (Figure 3).

## 3. Discussion

Phagocytes and macrophages in particular play an important role in clearing pathogens, apoptotic cells and cell debris. To investigate the in vitro effect of MIM on phagocytosis capacity, we have employed a platform that utilizes microscopy to monitor phagocytosis in real time, coupled with software able to measure the intracellular fluorescence only, thus eliminating background and false-positive results. MIM has increased the phagocytic activity of human primary macrophages (Figure 1B,C), starting from early time points, at both concentrations tested. The highest MIM concentration of 22 mM could have even overcome the effect of the phagocytosis inducer used as a control in this experiment. Even if no mechanistic assessment has been performed, we speculate that the two cytokines, TNF-α and IFN-γ, used in the tested MIM, respectively at 5 and 6 CH, could explain the enhanced phagocytic capacity of MIM-treated cells compared to vehicle-treated cells. It has been reported that phagocytosis and killing of *C. albicans* are enhanced by proinflammatory cytokines, in particular by TNF-α and IFN-γ [35]. The two cytokines may have worked synergistically, thus inducing autocrine positive feedbacks, as it was previously reported to happen with higher doses [20,21], finally boosting the phagocytic capacity. The recent publication of Calabrese et al. highlighted that very few molecules per cell are needed to effectively activate a cellular process via cascades of amplification mechanisms, and macrophages’ phagocytic capacity has been described as one of the documented physiological processes having extremely sensitive detection [36]. The authors showed that cAMP ranging from 10^−2^ to 10^−18^ M displayed a biphasic concentration-relationship over the macrophages’ phagocytic response, and the highest dilutions tested have induced a stimulation ranging between 125 and 150%, as compared to the control group.

Macrophages are important players in innate immunity, not only because of their phagocytic capabilities, but also because of their role as potent proinflammatory cytokine producers and antigen-presenting cells [37]. Macrophages have been identified in all tissues; they display remarkable plasticity and heterogeneity strictly dependent on their microenvironment. The classical activation of macrophages from the naïve status M0 to M1 is promoted by LPS, IFN-γ and GM-CSF. The resulting M1 phenotype is characterized by the secretion of proinflammatory cytokines that modulate/stimulate the Th1-mediated antigen-specific immune response. On the other side, M2 phenotypes can be induced by IL-4, IL-13, IL-10 and TGF-β and have pronounced anti-inflammatory properties [38]. In our model of human CD14^+^-derived macrophages, we found that both IFN-γ and MIM decreased the expression of CD16 and CD200R (Figure 2C). Our results are consistent with a previous study from Koning et al., who also assessed the expression of CD200R by flow cytometry and found a decrease in its expression in IFN-γ-treated macrophages [39]. Interestingly enough, in their transgenic model of CD200R^−/−^ mice, Goulding et al. showed that CD200R loss prevents *influenza*-induced secondary bacterial superinfection while promoting NK cells [40], which further strengthen the therapeutic potential of MIM as preventive care against respiratory infectious diseases. On the other side, when macrophages are stimulated with IL-10 (10 ng/mL), an M2a macrophage polarization inducer, their CD16 expression is reduced more than 3-fold [41]. Moreover, in our LPS-stimulated human CD14^+^-derived macrophages, MIM could slightly reduce the secretion of IL-10 (Figure 2D), an immunosuppressive cytokine playing a critical role in limiting the duration and the intensity of immune and inflammatory reactions. Interleukin-10 expression is tightly regulated, as excessive IL-10 can reduce the ability of immune responses to fight against infectious pathogens, while insufficient IL-10 is associated with chronic pathologic status, secondary to excessive tissue damage. During the active phase of an immune response characterized by an increase in IFN-γ production, IL-10 expression and activity can be restrained to efficiently clear the pathogens [42]. Coherently, IFN-γ has induced the suppression of this anti-inflammatory cytokine and the same trend has been observed in MIM-treated cells. The results presented here are undeniably not sufficient to conclude that MIM acts as an M1 macrophage polarization inducer in a similar manner to high doses of IFN-γ, but they reveal for the first time the potential of MIM to modulate macrophage phenotype, somewhat reflecting their possible engagement towards an M1-differentiated macrophage profile. These first pieces of evidence are definitely encouraging, considering the fact that the promotion of M1 macrophage polarization was also advocated as a strategy to inhibit IAV infections [43].

The in vivo part of the present study aimed at deciphering the potential of MIM as a preventive treatment against infectious diseases in a model of respiratory infection induced by IAV inoculation. Our results outlined the MIM effects in slightly increasing the neutrophil counts in the BALF as well as the circulating IgM levels after a 12-day treatment including 10 days of preventive treatment followed by 2 days of curative treatment post-IAV infection (Figure 3A,C,F). Accumulation of neutrophils at the site of inflammation is a typical mechanism of innate immunity. Those preliminary results are inspiring as they suggest that MIM could also modulate the adaptive immunity, characterized by the humoral response of the B cells. Despite the fact that no difference was found within the subtypes of circulating IgG between the treated and untreated groups, it is not impossible that other classes of Ig could have been impacted, out of the scope of this analysis. This hypothesis can be formulated because it is well known that the Ig switch is, at least partially, regulated by the IL-6 and IFN-γ secreted by the Th1 cells [44] and that those two factors are present in the formulation of MIM. As IgM has been shown to be the first responder to foreign invaders, including viral pathogens [45], and as it has been reported that *Influenza* virus neutralization depends on the presence of natural IgM in normal mouse serum [46], our results are encouraging as the effect of MIM on the humoral IgM response could be beneficial for the prevention and early treatment of respiratory infections. Interestingly, the effects of Citomix, another low-dose multicomponent-based medicine, on IgM secretion have previously been outlined in an ex vivo model of adenoidal mononuclear cells recovered from children who underwent operation for adenoid hypertrophy [47]. In this study, the tested product significantly increased the expression of B memory cells and IgM secretion, and it decreased IL-10. An interesting parallel with our work can be made as, even if their active ingredient formulations differ, Citomix and MIM share common low-dose cytokines (IFN-γ, IL-1β, IL-2 and IL-6) that may explain their biological effects as immune boosters.

PBMCs encompass immune players belonging to either innate or adaptive immune cell subtypes, all interacting together in the elaboration of the immune response. We assessed the ability of MIM to modulate the proliferation and the activation of immune cells in a naïve state, as well as in a context where the adaptive cells (i.e., the lymphocytes) would also be primed. The latter context might be likened to the situation occurring when immune responses to antigens are running and symptoms of viral or bacterial infections are already present. Immune cell activation models usually involve LPS, as these kinds of pathogen-associated molecular patterns (PAMPs) would interact with the pattern recognition receptors (PRR), leading to monocyte activation. However, as it was demonstrated that LPS treatment of PBMCs only affected CD14^+^ and did not lead to significant changes in PBMC composition [48], we used a more powerful model of T cell prepriming, involving the traditional use of anti-CD3 and anti-CD28 antibodies. As the activation of T cells through an anti-CD3 alone for 48 h increased the cell proliferation, IFN-γ production and IL-2R expression [49], we also used this “CD3-only” stimulating model to assess if MIM could potentially act as a costimulatory molecule and further support a stronger immune response in a preprimed immune state. We demonstrated here that MIM increased the proliferation of the total PBMCs, the NK cells and the T cells (CD4^+^ and CD8^+^) in both naïve and preprimed cells (Figure 4B–F), suggesting that its mode of action is independent of a T cell’s preactivation. It is also worth mentioning that, in presence of MIM, the proliferation of the T cells was even stronger than the one induced by the anti-CD3 alone (Supplementary Figure S2), suggesting that the active ingredients of this medicine could act as a potent costimulatory signal for those cells. Interestingly, it has previously been shown that the combination of anti-CD3 and IL-2 treatment induced the cell division of resting T cells, whereas such results were not obtained if the cells were incubated with the anti-CD3 antibody alone [28]. These data suggest that the capacity of MIM to stimulate the proliferation of T cells in presence of anti-CD3 alone could be attributable, at least partially, to the presence of IL-2 (at 10 CH) in the tested capsule (Figure 4D–F and Figure S2). Human CD69 differentiation antigen is one of the earliest cell surface molecules expressed after activation of lymphocytes and hematopoietic cells, including NK cells [50]. In addition to confirming the data from Lawlor et al., who demonstrated that PBMCs stimulated with anti-CD3/CD28.2 resulted in an indirect activation of NK cells [48], our results showed that, in a preprimed immune state, MIM can considerably exacerbate the expression of this activation marker in NK and in CD8^+^ T cells (Figure 4G,H and Figure S3). CD69 upregulations have been shown in response to IFN-γ treatment in eosinophil precursors [51] and consecutively to a TNF-α treatment in erythroleukemic K562 cells, as it was also demonstrated that CD69 promoter contains TNF-α-responsive elements [52]. Indeed, the effect of MIM in increasing the expression of CD69 could be attributable, at least partially, to the presence of IFN-γ (6 CH) and TNF-α (5 CH) in the tested MIM capsule. Interestingly, as the phagocytic capabilities of NK cells are correlated with their activation status characterized by an increase in CD69 expression [53], our findings are coherent and also suggest that MIM could potentiate the phagocytosis of these cells and sustain immune responses and host defenses against pathogens. Finally, the effects of MIM in activating monocytes/macrophages through increasing the expression of HLA-DR (Figure 4I and Figure S4) could be, at least partially, attributable to the presence of IFN-γ (6 CH), as it has been shown that IFN-γ increased the expression of HLA-DR in monocytes [54].

Endothelial cells are fine-tuners of the immune responses as they share common innate immune functions with macrophages, such as cytokine secretion, antigen presentation, PAMP sensing or even phagocytosis, and their plasticity allows them to regulate the immune surveillance over the different tissues of the organism [30]. Coherently with previous findings [31,32,33,34], TNF-α alone is able to upregulate the four tested endothelial markers. Furthermore, all the cytokines used in MIM are shown to stimulate those markers [55,56,57,58]. Interestingly, we report here that MIM can upregulate, even if just slightly, the expression of five endothelial cells markers related to immune response modulation (Figure 5), similar to the proinflammatory cytokine TNF-α. The increase in the HLA-I expression may be linked to the immune cell recruitment observed in vivo (Figure 3C), as HLA-I signalization was reported to impact leukocyte tethering or neutrophil adherence for instance [59]. The slight increase in the CD137L expression can contribute to the immune-boosting effect of MIM, as CD137L signaling in endothelial cells plays a crucial role in the production of proinflammatory cytokines and chemokines implicated in the activation and the recruitment of neutrophils [60]. Moreover, the CD137/CD137L interaction not only constitutes a costimulatory signal triggering the expansion of the CD4^+^ and CD8^+^ T cells, but also amplifies the inflammatory responses mediated by NK cells [61]. Our results also showed that MIM tends to increase the expression of GITRL on the HUVEC surface (Figure 5), which may potentially facilitate GITR/GITRL signaling and thus result in T cell activation and proliferation [62]. Indeed, numerous studies using suboptimal preprimed anti-CD3 stimulated T cells reported the role of the GITR/GITRL axis as a costimulation signal for the T cells [63]. Moreover, it has also been reported that GITR interaction stimulates antiviral responses through enhanced TNF-α and IFN-γ production in vivo, which may also, at least partially, contribute to explaining the systemic effects of MIM observed in the murine model of viral infectious disease (Figure 3) [64]. Although this first body of data supports the fact that MIM tends to stimulate the expression of endothelial cells markers triggering the proliferation/activation of T cells, our results also showed that MIM increased the expression of the checkpoint inhibitor PD-L1. As IFN-γ was shown to promote PD-L1 expression to prevent the overactivation of T cells, thus allowing the inflamed tissues to be protected against an excessive immune activity [65,66], the presence of IFN-γ (6 CH) in the tested capsule may explain the observed results. As ICAM-1 is implicated in the firm adhesion of the activated T cells to the endothelial cells, as well as in their transendothelial migration, our results finally suggest that MIM could sustain the recruitment of lymphocytes to the inflammation site [67]. The expression of ICAM-1 is induced by TNF-α, IL-1β [68] and IL-6 [69], and all of these signaling molecules are present in MIM (5, 6 or 10 CH). Interestingly, ICAM-1 was also shown to regulate IAV during the early stage of infection, and ICAM-1 silencing was associated with an increase in the *influenza* matrix gene copy number [70]. Those results could thus reinforce the explanation of the systemic effects of MIM observed in this study.

## 4. Materials and Methods

### 4.1. Tested Items

MIM, provided by Labo’Life Belgium, is a homeopathic medicinal product presented in a form of sequential treatment, consisting of sucrose–lactose pilules, also termed globules, impregnated with ethanolic preparations of LDs and/or ULDs of immune mediators (interleukins and growth factors) and nucleic acids (DNA, RNA and SNA). The pilules are enclosed into capsules, which are then packaged into blisters. The composition of the entire MIM sequence is indicated in Table 1 (middle column). The entire MIM sequence was tested in vivo and administered to the mice, according to the order indicated in blister (from 1 to 10). Due to confidentiality issues arising from intellectual property, the composition of each specific capsule has not been disclosed. Labo’Life Belgium also manufactured and provided the vehicle pilules, the experimental control used in all the in vitro and in vivo studies.

The vehicle, also named Veh. in the article, consists of sucrose–lactose pilules impregnated with the same ethanolic preparation used to prepare LDs and/or ULDs, but lacking active substances (see Supplementary Figure S1, which illustrates the Veh. production in parallel with an example of MI medicine).

Regarding the in vitro experiments, only one capsule of the sequence was tested. The composition of this capsule is as follows: hr-IL-1β (10 CH), hr-IL-2 (10 CH), hr-IL-5 (10 CH), hr-IL-6 (10 CH), hr-IFN-γ (6 CH), hr-TGF-β (10 CH), hr-TNF-α (5 CH), DNA (10 CH), RNA (10 CH), SNA-HLA I (10 CH), SNA-HLA II (10 CH) and SNA-EID (10 CH) (see Table 1, right column).

### 4.2. Phagocytosis Capability Assessment Experiments

Peripheral blood mononuclear cells (PBMCs) from healthy volunteers were obtained from Etablissement Français du Sang (EFS) after being isolated from buffy coats and standard Ficoll-Hypaque gradient method. Monocytes were isolated from PBMCs by adherence to plastic for 2 h in serum-free macrophage-SFM medium (M-SFM, Gibco, Life Technologies, Carlsbad, CA, USA) optimized for macrophage culture, at 37 °C in a humidified atmosphere containing 5% CO_2_. Monocytes were seeded and differentiated in macrophages in presence of granulocyte macrophage colony-stimulating factor (GM-CSF) and IFN-γ, provided by Peprotech, Inc. Cranbury, NJ, USA. Regarding *C. albicans* labeling protocol, briefly, *C. albicans* was heat-killed (1 h at 75 °C) before labeling with pHRodo Red, succinimidyl ester (pHRodo Red, SE, ThermoFisher Scientific, Waltham, MA, USA), according to the manufacturer’s instructions. The pHRodo dye is known as a nonfluorogenic molecule at neutral pH, drastically becoming fluorescent in acidic milieu. This situation happens in phagolysosomes, thus indicating effective phagocytosis. *C. albicans* was introduced in sodium bicarbonate with pHRodo dye (final concentration of 20 μg/mL) for 1 h, RT, in the dark. Excess dye was washed with phosphate-buffered saline (PBS) solution. Labeled *C. albicans* was finally diluted in PBS and left at 4 °C until use. Control for correct labeling was realized in an acid solution (pH = 4) vs. neutral solution (pH = 7.4). After one week of monocyte differentiation into macrophages, MIM and vehicle were incubated for 24 h with the cells at two different sucrose–lactose concentrations: 11 and 22 mM. A positive control (not disclosed and kept confidential by the contract research organization (CRO) that conducted the study) was also included in the experiment. The next day, culture medium and its treatments were replaced, before pHRodo-labeled *C. albicans* was added to the cells and left on ice to allow the proper sedimentation of *C. albicans*. After 30 min on ice, the plate was read on an Operetta apparatus for 6 h at 37 °C, 5% CO_2_, and data acquisition was realized thanks to Harmony Imaging Software (Perkin Elmer France, Villebon-sur-Yvette, Every, France). Each well was imaged every 8 min and the Columbus 2.5.0 image analysis software was used to conduct the phagocytosis quantitation. The experimental scheme is described in Figure 1A. The experiment was carried out once and six replicates were run for all conditions; indeed, no statistical inference was performed according to the recommendation in terms of statistical analysis in fundamental research [71].

### 4.3. Macrophage Cell Surface Marker Expression and Cytokine Secretion Evaluation

CD14^+^ monocytes were freshly isolated from PBMCs thanks to the Miltenyi kit (130-050-201). Briefly, the cells were then seeded at the density of 50,000 cells/well in 96-well plates on Day 0 and were cultivated in RPMI 1640 supplemented with 2% inactivated human serum, 1 mM nonessential amino acids, 1 mM pyruvate, 2 mM L-glutamine, 10 mM HEPES buffer and 50 ng/mL M-CSF (also known as CSF-1). Cells were treated with MIM/vehicle (at 5.5 or 11 mM) or IFN-γ 50 ng/mL (M1 macrophage-differentiation inducer) from Day 1 until Day 7, both medium and treatments being renewed on Day 3 and Day 5. The experimental scheme is shown in Figure 2A.

For the macrophage cell surface marker experiment, the cells were harvested, labeled and analyzed by flow cytometry on Day 7 on a BD FACS Canto II, configuration 4/2/2. The intensity of the staining was measured as median fluorescence intensity (MFI) value. The cell viability was appraised based on a gating of the NIR-Zombie positive cells. The M0 and M1 macrophage populations were discriminated based on their expression of CD14/CD64/CD86 and identified as follows: M0: CD14^+/−^, CD64^+^, CD86^+^; M1: CD14^++^, CD64^+++^, CD86^++^. The expression of the cell surface markers CD16 and CD200R was also evaluated. For cytokine secretion assessment, the cells were stimulated with lipopolysaccharide (LPS) on Day 6 (100 ng/mL) and harvested on Day 7. Supernatants were collected after cell centrifugation, and cytokine measurement was directly performed by ELISA (LegendPlex), on fresh supernatants. The secretion of the anti-inflammatory cytokine IL-10 was analyzed. The secretion levels of TNF-α and IFN-γ were measured as well. Because only one experiment was conducted, no statistical inference has been performed. The results of the viability, cell markers and IL-10 secretion are presented as mean ± SD of n = 3 replicates per condition.

### 4.4. In Vivo Experiments

The in vivo experiment was conducted in ArtImmune/CNRS (TAAM UPS44, Orléans, France) animal facility, in compliance with the guidelines of the French Ministry of Agriculture for experiments with laboratory animals (Law 87-848) and with Animal Health regulations. All animals were kept on a 12 h light–dark cycle, at a controlled temperature (22 ± 1.5 °C) and 50 ± 25% hygrometry, and were housed in solid-bottomed cages with chip bedding. The animals were provided with free access to standard food and water. The protocol was submitted to the Ethics Committee for Animal Experimentation of CNRS Campus Orléans (CCO) and approved by the French Minister under number APAFIS #19230.

The respiratory infection model was induced on Day 0 by intranasal administration of IAV H3N2 (Scotland/20/74) strain in 9-week-old female BALB/cJRj mice (n = 10 mice per group) purchased from Janvier, France. Mice were infected intranasally with 40 µL of saline solution containing 100 PFU of IAV under a mixture of ketamine–xylazine (1 and 0.2 mg/mouse, respectively) or saline solution alone (control group). Preventive treatments with MIM or sucrose–lactose vehicle pills were given daily from Day –10 to Day 2 by oral gavage route. Briefly, each day, the content of one capsule of MIM or control (Veh.) was freshly diluted in 25 mL of sterile ultrapure water, and 2500 µL of this solution was then diluted into 7500 µL of ultrapure water to reach the concentration of 11 mM. Each mouse finally received daily 200 µL of 11 mM diluted pilules, corresponding to 0.75 mg/mouse. Mice were observed daily for signs of morbidity, and blood was collected on Day 0, before infection. Mice were weighed daily for body weight changes and euthanized on Day 3 for further analysis. Briefly, total and differential cell counts were assessed in BALF, viral load was analyzed in lung homogenates and immunoglobulin levels were analyzed in plasma. The experimental scheme is illustrated in Figure 3A.

### 4.5. Blood Collection

Blood from all animals included in the experiment was drawn at the inferior vena cava into tubes containing lithium–heparin (as an anticoagulant). Tubes were centrifuged 5 min at 5000 rpm at 4 °C and plasma was collected, aliquoted and frozen at −80 °C for further analysis.

### 4.6. Plasmatic Concentrations of Immunoglobulins Assessment

IgG1, IgG2a, IgG2b, IgG3 and IgM levels in plasma were determined by Luminex immunoassay according to the manufacturer’s recommendations (#MGAMMAG-300K-05) and by using MagPix reader (Bio-Rad Laboratories, Hercules, CA, USA). Results were reported as pg/mL.

### 4.7. Bronchoalveolar Lavage Fluid Differential Leukocyte Counts

The lungs were washed four times with 0.5 mL of saline solution at room temperature. After centrifugation at 400 g for 10 min at 4 °C, supernatants of the first lavage (cell-free BALF) were stored at −80 °C for cytokine dosage. An aliquot of the cell pellets was stained with Turk’s solution and counted, and 200,000 cells were centrifugated onto microscopic slides (Cytospin at 1000 rpm for 10 min, RT). Air-dried preparations were fixed and stained with Diff-Quick (#130832, Medion Diagnostics AG, Merz & Dade, Berlin, Germany). Differential counts were performed using oil immersion light microscopy. One hundred cells were observed twice in light microscopy for the determination of percentage and the absolute number of the differential cell count.

### 4.8. Viral Load Determination

Lung tissues were homogenized in Trizol (Tri-Reagent, #T9424, Sigma-Aldrich, Saint-Louis, MO, USA) and total mRNA was extracted using phenol–chloroform protocol. Briefly, lung tissues were ground into phenol–chloroform (0.1 mL/0.2 mL) and vigorously mixed. After centrifugation (15 min at 12,000 rpm, 4 °C), the transparent upper phase containing the RNA was gently collected and mixed with 0.5 mL of isopropanol and incubated for 15 min on ice. The solution was further centrifuged (10 min at 12,000 rpm, 4 °C), the supernatant was discarded and the pellet was washed twice in 1 mL of 70% ethanol, followed by another centrifugation (5 min at 7500 rpm, 4 °C). Pellets were finally dried and suspended in 30 μL of sterile ultrapure water. cDNA was obtained from 1 μg of total RNA by reverse transcription using GoScript Reverse Transcriptase and random primers (A5001, GoScript Reverse Transcription System, Promega, Madison, WI, USA) according to the manufacturer’s instructions (Promega). The reverse transcription program consisted of 5 min at 25 °C, 60 min at 42 °C and 15 min at 70 °C. Quantitative PCR was conducted using an AriaMx real-time quantitative PCR analysis with GoTaq qPCR Master Mix 2 × (A6002, Promega) with 10 μM of forward and reverse primers (Flu-1: 5′-AAGACCAATCCTGTCACCTCTGA-3′; #HA05057218, Flu-2: 5′-CAAAGCGTCTACGCTGCAGTCC-3′; #HA05057219, Sigma). The PCR program consisted of 10 min at 95 °C for polymerase activation; 40 cycles of 30 s at 95 °C for DNA denaturation, 30 s at 60 °C for primer annealing and 30 s at 72 °C for primer extension; and 1 min at 55 to 95 °C for the progressive increase in temperature for the generation of the dissociation curve. Viral RNA load was expressed in absolute number. Titer was determined from a standard curve which was prepared from dilution series of IAV H3N2 Scotland/20/74 virus strain of known concentration. The standard curve approach also named “absolute quantification” was used to measure the exact level of viral load in the sample. Following amplification of the standard dilution series, the standard curve was generated by plotting the log of the initial viral copy number against the Ct generated for each dilution. These points generated a linear regression line in which the Ct values of the unknown samples were compared in order to quantify the initial copy number.

### 4.9. Evaluation of Cell Surface Activation Marker Expression and Proliferation of PBMC Sub-Populations by Flow Cytometry

Freshly isolated PBMCs were cultivated in RPMI 1640 medium supplemented with 2% inactivated human serum, 1 mM nonessential amino acids, 1 mM pyruvate and 10 mM HEPES buffer, in presence or absence of 0.5 µg/mL bottom-coated OKT3 antibody (CD3); and combined or not with 2 µg/mL of soluble anti-CD28.2 (CD3 + CD28.2). Concanavalin A (5 µg/mL), which is able to induce the expression of CD69, was used as a positive control. Immune cell populations were delineated based on the following panels in the proliferation experiment: natural killers (NK): CD3^−^, CD11b^−^, CD4^−^, CD8^−^, CD19^−^, CD56^+^, CD14^−^, SSC^low^; monocytes/macrophages: CD3^−^, CD11b^+^, CD4^−^, CD8^−^, CD19^−^, CD56^−^, CD14^+^, SSC^low^; granulocytes: CD3^−^, CD11b^+^, CD4^−^, CD8^−^, CD19^−^, CD56^−^, CD14^−^, SSC^high^; B cells: CD3^−^, CD11b^−^, CD4^−^, CD8^−^, CD19^+^, CD56^−^, CD14^−^, SSC^low^; T cells: CD3^+^, CD11b^−^, CD4^+^, CD8^+^, CD19^−^, CD56^−^, CD14^−^, SSC^low^; CD4^+^: CD3^+^, CD11b^−^, CD4^+^, CD8^−^, CD19^−^, CD56^−^, CD14^−^, SSC^low^; CD8^+^: CD3^+^, CD11b^−^, CD4^−^, CD8^+^, CD19^−^, CD56^−^, CD14^−^, SSC^low^. In the NK cell activation experiment, the CD69 expression was assessed amongst the NK population characterized by the following panel: CD3^−^, CD11b^−^, CD4^−^, CD8^−^, CD19^−^, CD56^+^, SSC^low^. In the monocyte/macrophage activation experiment, the HLA-DR expression was assessed amongst the population characterized by the following panel: CD3^−^, CD11b^+^, CD14^+^, SSC^low^.

### 4.10. HUVEC Immunoprofiling by Flow Cytometry

Primary human umbilical vein endothelial cells (HUVECs) (Passage 4) were seeded at 5000 cells/well in a 96-well plate in ECGM medium (PromoCell, Heidelberg, Germany) supplemented with 2% FBS and were grown for 4 days before adding MIM/vehicle to the medium at the desired concentrations. The cells were treated for 48 h before multiparametric immunostaining and flow cytometry analysis. The intensity of the staining was measured as median fluorescence intensity (MFI) value. One experiment was performed in triplicate for each condition. The viability and the expression levels of HLA-I, CD137 ligand (CD137L), glucocorticoid-induced tumor necrosis factor receptor-related protein ligand (GITRL), programmed death-ligand 1 (PD-L1) and intercellular adhesion molecule-1 (ICAM-1) were assessed.

### 4.11. Statistical Analysis

The graphs in figures were created with GraphPad Prism, Version 9 for Windows (GraphPad Software Inc., San Diego, CA, USA, www.graphpad.com, accessed on 29 July 2021). Authors have followed the recent recommendations that encourage performing descriptive statistics instead of making statistical inferences when the number of independent values is small. Indeed, no statistical inference has been performed to analyze the results of the in vitro studies. In order to show the results in a transparent manner, when data were obtained from only one experiment, replicates were plotted together with the mean ± SD in the graphs (Figure 1, Figure 2 and Figure 5).

In the PBMC experiments, the data are represented as the mean ± SEM of values obtained from n = 3 healthy donors (Figure 4, Figures S2–S4). Concerning the in vivo study, statistical evaluation of differences between the experimental groups was determined for different parameters: (i) body weight, analyzed by using two-way ANOVA followed by a Dunnett’s multiple comparisons post-test; (ii) neutrophil counts in BALF on D3, analyzed by nonparametric Mann–Whitney test; (iii) plasmatic immunoglobulin levels of IgM on D3, analyzed by nonparametric Mann–Whitney test; (iv) correlations between the viral load in the lungs and the number of neutrophils in the BALF on D3 in the vehicle- and MIM-treated groups, evaluated by Pearson’s r scores. *p* values of less than 0.05 were considered significant.

## 5. Conclusions

Overall, the present study shows that MIM acts as an immune enhancer towards several actors of both the innate and the adaptive immune system, implicated in the organism’s defense. The in vivo mice model of *influenza*-induced respiratory disease highlighted the potential beneficial effects of MIM against viral-mediated respiratory diseases as a preventive and early treatment, as MIM acted at (i) a local level, in promoting the recruitment of neutrophils to the lungs, and (ii) at a systemic level, in increasing the circulating levels of IgM in the treated animals, compared with the control group. This integrated model of immunocompetent mice provided the proof of concept that MIM could act on both sides of the innate and the adaptive immune responses. Those preliminary results were confirmed in vitro, by using human PMBCs isolated from healthy volunteers, in which MIM increased the proliferation of total cells, NK cells and CD4^+^ and CD8^+^ T cells, independently of the setting of a preprimed T cell context. Moreover, the activation state of the NK cells, the CD8^+^ T cells and the monocytes/macrophages was also enhanced, as evidenced by the increase in CD69 and HLA-DR expression, suggesting that MIM could act as a costimulatory signal for those cells. We also reported here that MIM especially acts on the innate immune responses as it increased the phagocytic activity of macrophages and modulated their cell surface expression of CD16 and CD200R and their IL-10 cytokine secretion in a pattern somewhat similar to that of the IFN-γ-derived M1 macrophages. HUVEC profiling finally revealed that MIM allowed a fine modulation of the expression of some immune-related markers such as HLA-I, CD137L, GITRL, PD-L1 and ICAM-1. These results contribute to a better understanding of MIM’s holistic mode of action, bridging the gap between the local and the systemic effects of this medicine found in vivo. In conclusion, the present study suggests that MIM might be a promising nonspecific (without antigen specificity) immunostimulant drug for the prevention and early treatment of respiratory infections, but not only exclusively, as it would gently support several facets of the immune system, sustaining host defenses against pathogens.

## Figures and Tables

**Table 1:** Composition of the complete sequence of the MIM (in vivo experiment) and the tested capsule (in vitro experiments).

Ingredients (CH)	MIM Entire Sequence	MIM Tested Capsule
hr-IL-1β	5–10	10
hr-IL-2	5–10	10
hr-IL-5	6–10	10
hr-IL-6	6–10	10
hr-IFN-γ	6–10	6
hr-TGF-β	30–10	10
hr-TNF-α	5–10	5
DNA	8–10	10
RNA	8–10	10
SNA-HLA I	10	10
SNA-HLA II	16–10	10
SNA-EID	16–10	10

Each active ingredient is given in CH. CH: centesimal Hahnemannian dilutions; SNA: specific nucleic acids.

## Data Availability

Not applicable.

## Supplementary Materials

The following are available online at https://www.mdpi.com/article/10.3390/ijms23010110/s1.

## Abbreviations

AA: amino acids; BALF: bronchoalveolar lavage fluid; CD137L: cluster of differentiation 137 ligand; CH: centesimal Hahnemannian; GITRL: glucocorticoid-induced tumor necrosis factor receptor-related protein ligand; GM-CSF: granulocyte macrophage colony-stimulating factor; HLA: human leukocyte antigen; HUVEC: human umbilical vein endothelial cell; IAV: *influenza A* virus; ICAM-1: intercellular adhesion molecule-1; IFN-γ: interferon-γ; Ig: immunoglobulin; IL: interleukin; i.n.: intranasal; LD: low dose; LPS: lipopolysaccharide; M1: M1 macrophage; M-CSF: macrophage colony-stimulating factor; MI: micro-immunotherapy; MIM: micro-immunotherapy medicine; NK: natural killer; PAMP: pathogen-associated molecular pattern; PBMCs: peripheral blood mononuclear cells; PBS: phosphate-buffered saline; PD-L1: programmed death-ligand 1; PRR: pattern recognition receptor; SNA: specific nucleic acids; TGF-β: transforming growth factor-β; TNF-α: tumor necrosis factor-α; ULD: ultralow dose.
