# Supplementary material for: The Micro-Immunotherapy Medicine 2LEID Exhibits an Immunostimulant Effect by Boosting Both Innate and Adaptive Immune Responses
Source: Int J Mol Sci. 2021 Dec 22;23(1):110. doi: 10.3390/ijms23010110 (PMC8744989; doi:10.3390/ijms23010110)
Supplement: Supplementary file 1 [file ijms-23-00110-s001.zip › paper 2LEID Sup Fig Legends_revised.pdf]

**Sup Figure S1: Representative scheme of the production of a MI medicine example and the Vehicle (Veh.).** The produced MI medicine given in this example contains two human recombinant (hr) cytokines, cytokine-A and cytokine-B, respectively prepared at the final CH of 5 CH and 4 CH. Starting from the initial "starting concentration", the first 1/100 dilution of the cytokines is made in $H_2O$, and the following, in 96% (v/v) ethanol (EtOH). The "sequential kinetic process" (SKP) consists of one 1/100 dilution followed by a vertical shaking step. The SKP is repeated until reaching the desired CH for each cytokine included in the final formulation of the desired MI medicine. The production of the Veh. globules is also schematically represented. Regarding the orange color used to represent the globules of MI medicine, this is simply for the purpose of a better graphic understanding, as all globules (of both Veh. and MI medicines) are white.

**Sup Figure S2: MIM increases the proliferation of PBMCs *in vitro*.** Human PBMCs from three healthy donors (referred as donors #A, #B and #C) were cultivated during 48 hours in (i) classical culture condition (Ct medium thereafter referred as "No signal") or in (ii) classical culture conditions plus coated anti-CD3 antibody at 0.5 µg/mL (thereafter referred as "anti-CD3"), or in (iii) classical culture conditions plus coated anti-CD3 antibody at 0.5 µg/mL + soluble anti-CD28 antibody at 2 µg/mL (thereafter referred as "anti-CD3 + anti-CD28") in presence of MIM/Veh. at 11 mM. Concanavalin A (5 µg/mL) was used as a positive control, regarding its stimulator's effects towards the induction of the CD69 expression (See **Sup Fig. 3**). The total number of cells and the total cell count within each subpopulation (NK, LT, $CD4^+$ and $CD8^+$ T cells) were assessed by flow cytometry. The total number of cells **(A)**, the NK cells count **(B)**, the T cells count **(C)**, the $CD4^+$ T cells count **(D)** and the $CD8^+$ T cells count **(E)** were evaluated in the conditions defined in (i) (**left panels**), (ii) (**middle panels**) and (iii) (**right panels**). Each cell subpopulation was differentiated from the other PBMCs according to the expression of the markers referred in the Material and Methods section. The experiment has been done once and each histogram represents the mean ± SEM of the cell count of technical triplicates obtained for each individual donor.

**Sup Figure S3: MIM increases the expression of the activation markers CD69 in NK cells and $CD8^+$ T cells *in vitro*.** Human PBMCs from three healthy donors (referred as donors #A, #B and #C) were cultivated during 48 hours in (i) classical culture condition (Ct medium thereafter referred as "No signal") or in (ii) classical culture conditions plus coated anti-CD3 antibody at 0.5 µg/mL (thereafter referred as "anti-CD3"), or in (iii) classical culture conditions plus coated anti-CD3 antibody at 0.5 µg/mL + soluble anti-CD28 antibody at 2 µg/mL (thereafter referred as "anti-CD3 + anti-CD28") in presence of MIM/Veh. at 11 mM. Concanavalin A (5 µg/mL) was used as a positive control, regarding its stimulator's effects towards the induction of the CD69 expression. The CD69 expression was assessed by flow cytometry within the NK cells **(A)** and the $CD8^+$ T cells **(B)** subpopulations in the conditions defined in (i) (**left panels**), (ii) (**middle panels**) and (iii) (**right panels**). Each cell subpopulation was differentiated from the other PBMCs according to the expression of the markers referred in the Material and Methods section. The experiment has been done once and each histogram represents the mean ± SEM of the CD69 expression in technical triplicates obtained for each individual donor.

**Sup Figure S4: MIM increases the expression of the activation marker HLA-DR in monocytes/macrophages *in vitro*.** Human PBMCs from three healthy donors (referred as donors #A, #B and #D) were cultivated during 48 hours in (i) classical culture condition (Ct medium thereafter referred as "No signal") or in (ii) classical culture conditions plus anti-CD3 antibody at 0.5 µg/mL (thereafter referred as "anti-CD3"), or in (iii) classical culture conditions plus anti-CD3 antibody at 0.5 µg/mL + anti-CD28 antibody at 2 µg/mL (thereafter referred as "anti-CD3 + anti-CD28") in presence of MIM/Veh. At 11 mM. The HLA-DR expression was assessed by flow cytometry within the monocytes/macrophages cells in the conditions defined in (i) (**left panels**), (ii) (**middle panels**) and (iii) (**right panels**). The monocyte/macrophage subpopulation was differentiated from the other PBMCs according to the expression of the markers referred in the Material and Methods section. The experiment has been done once and each histogram represents the mean ± SEM of the HLA-DR expression in technical triplicates obtained for each individual donor.
