# Supplementary material for: The Micro-Immunotherapy Medicine 2LEID Exhibits an Immunostimulant Effect by Boosting Both Innate and Adaptive Immune Responses
Source: Int J Mol Sci. 2021 Dec 22;23(1):110. doi: 10.3390/ijms23010110 (PMC8744989; doi:10.3390/ijms23010110)
Supplement: Supplementary file 1 [file ijms-23-00110-s001.zip › Sup Figure S4.pdf]

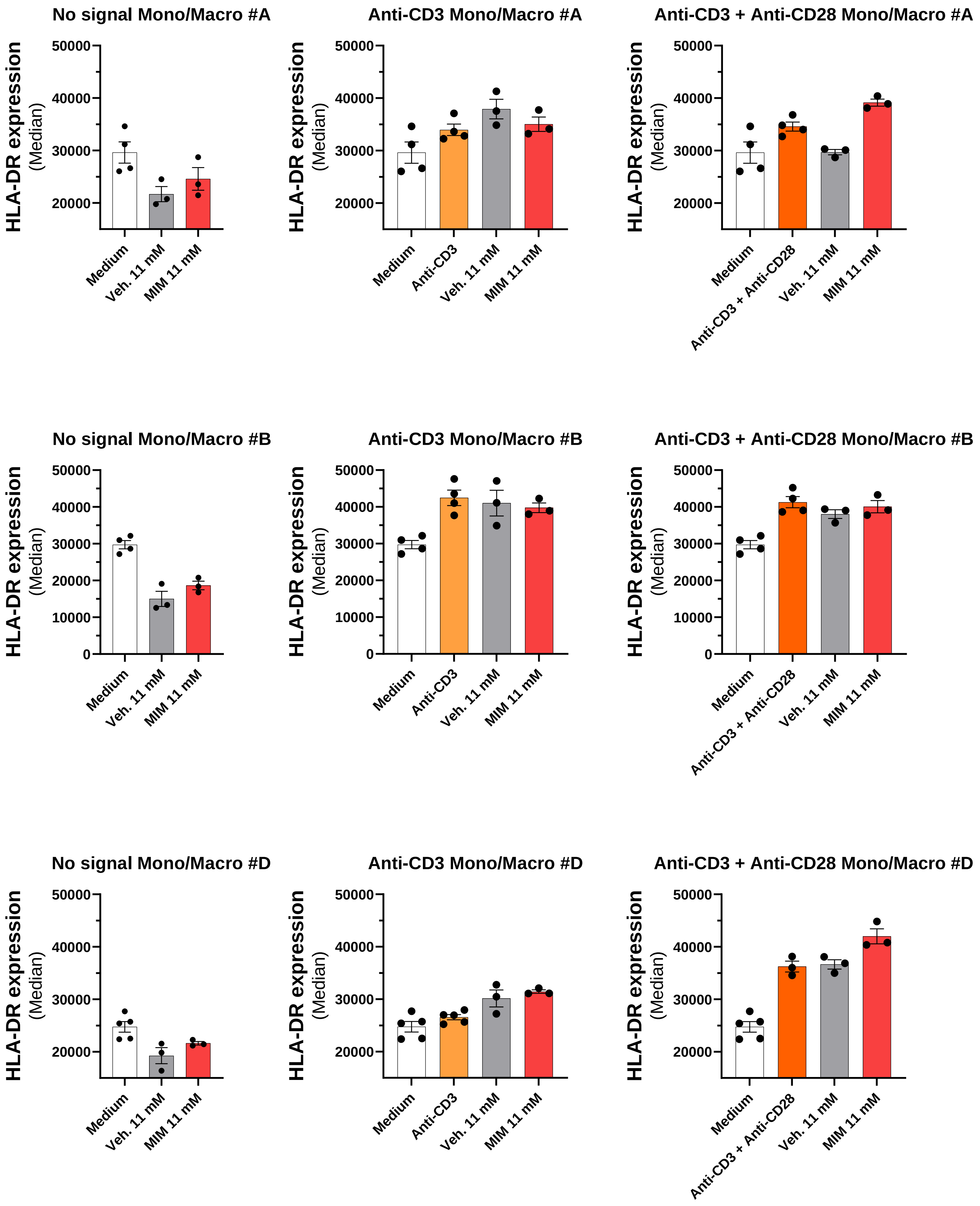
No signal Mono/Macro #A
Anti-CD3 Mono/Macro #A
Anti-CD3 + Anti-CD28 Mono/Macro #A
No signal Mono/Macro #B
Anti-CD3 Mono/Macro #B
Anti-CD3 + Anti-CD28 Mono/Macro #B
No signal Mono/Macro #D
Anti-CD3 Mono/Macro #D
Anti-CD3 + Anti-CD28 Mono/Macro #D
HLA-DR expression
(Median)
0
10000
20000
30000
40000
50000
Medium
Anti-CD3
Anti-CD3 + Anti-CD28
Veh. 11 mM
MIM 11 mM
