# Supplementary figures and images for: The Micro-Immunotherapy Medicine 2LEID Exhibits an Immunostimulant Effect by Boosting Both Innate and Adaptive Immune Responses
Source: Int J Mol Sci. 2021 Dec 22;23(1):110. doi: 10.3390/ijms23010110 (PMC8744989; doi:10.3390/ijms23010110)

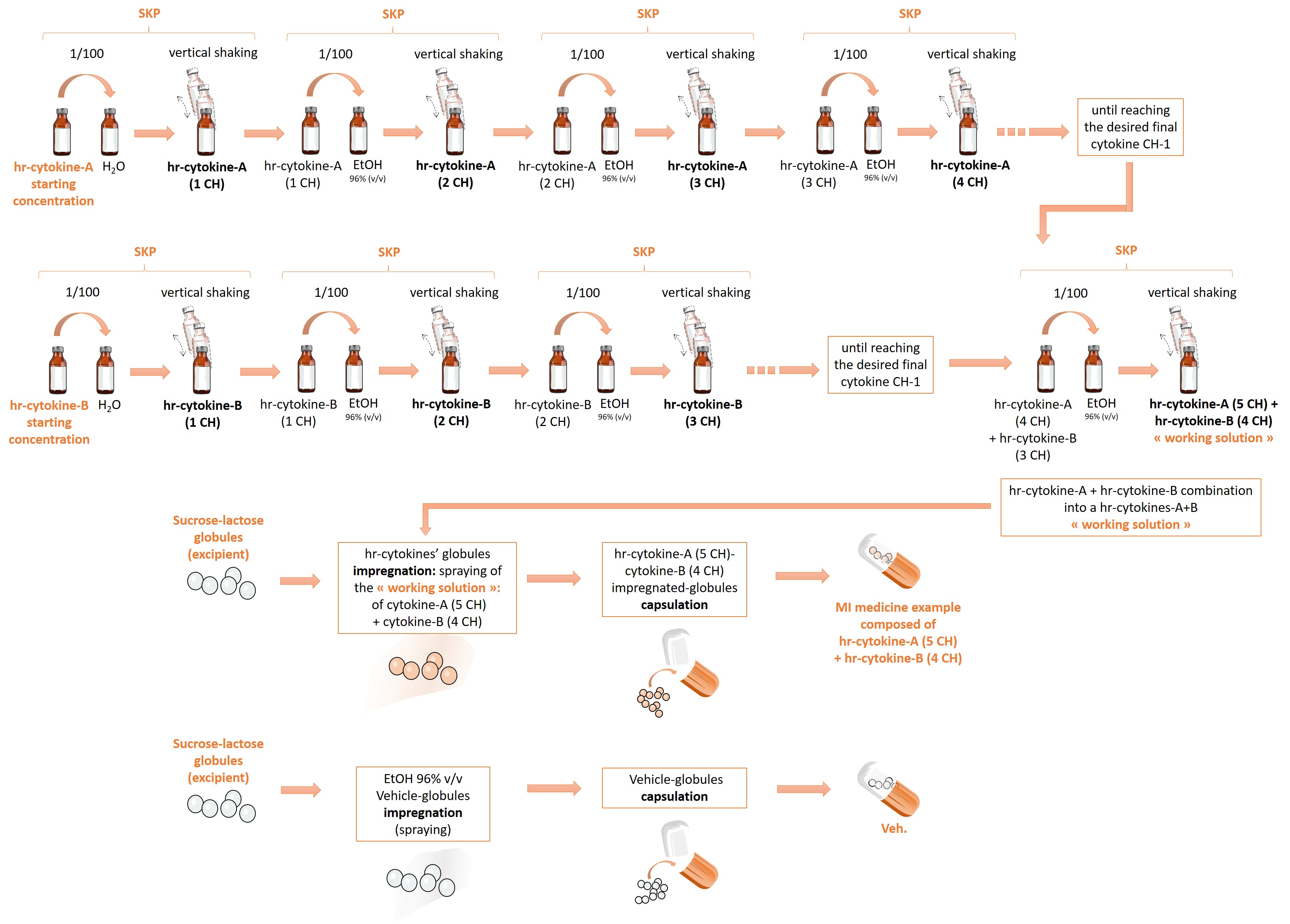

Supplement: Supplementary file 1 [file ijms-23-00110-s001.zip › Sup Figure S1.jpg]

**A**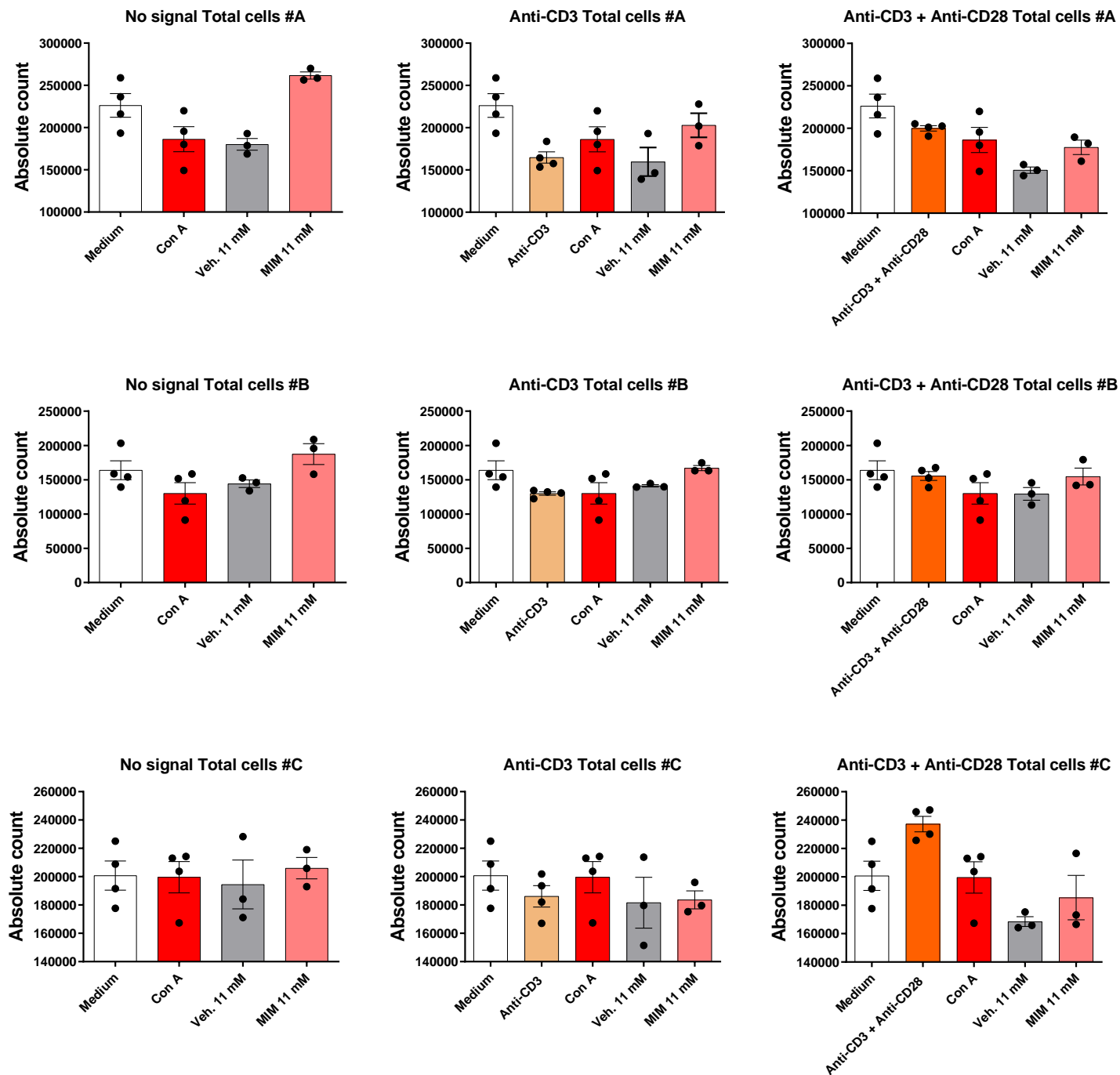

**B**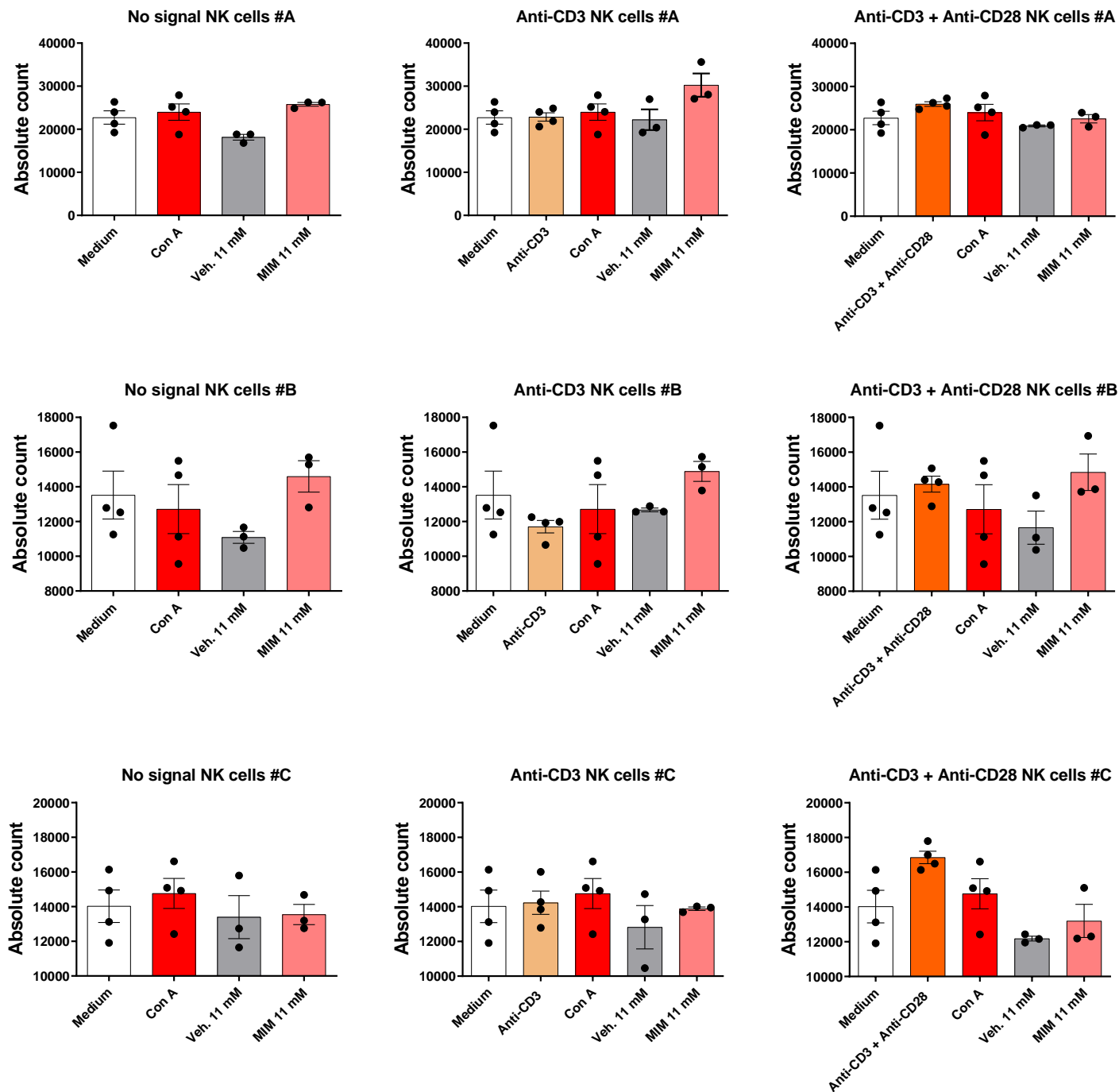

C

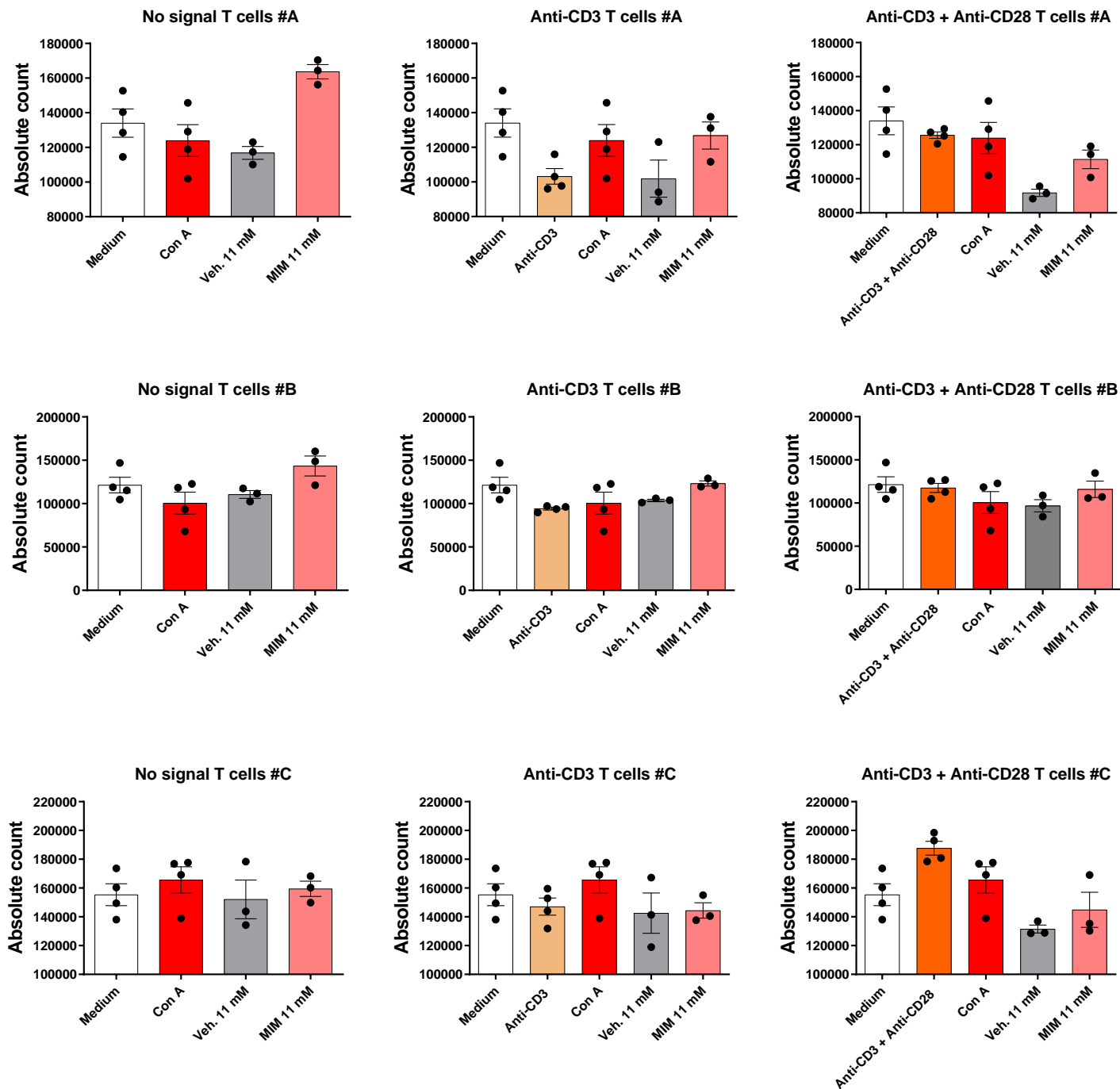

D

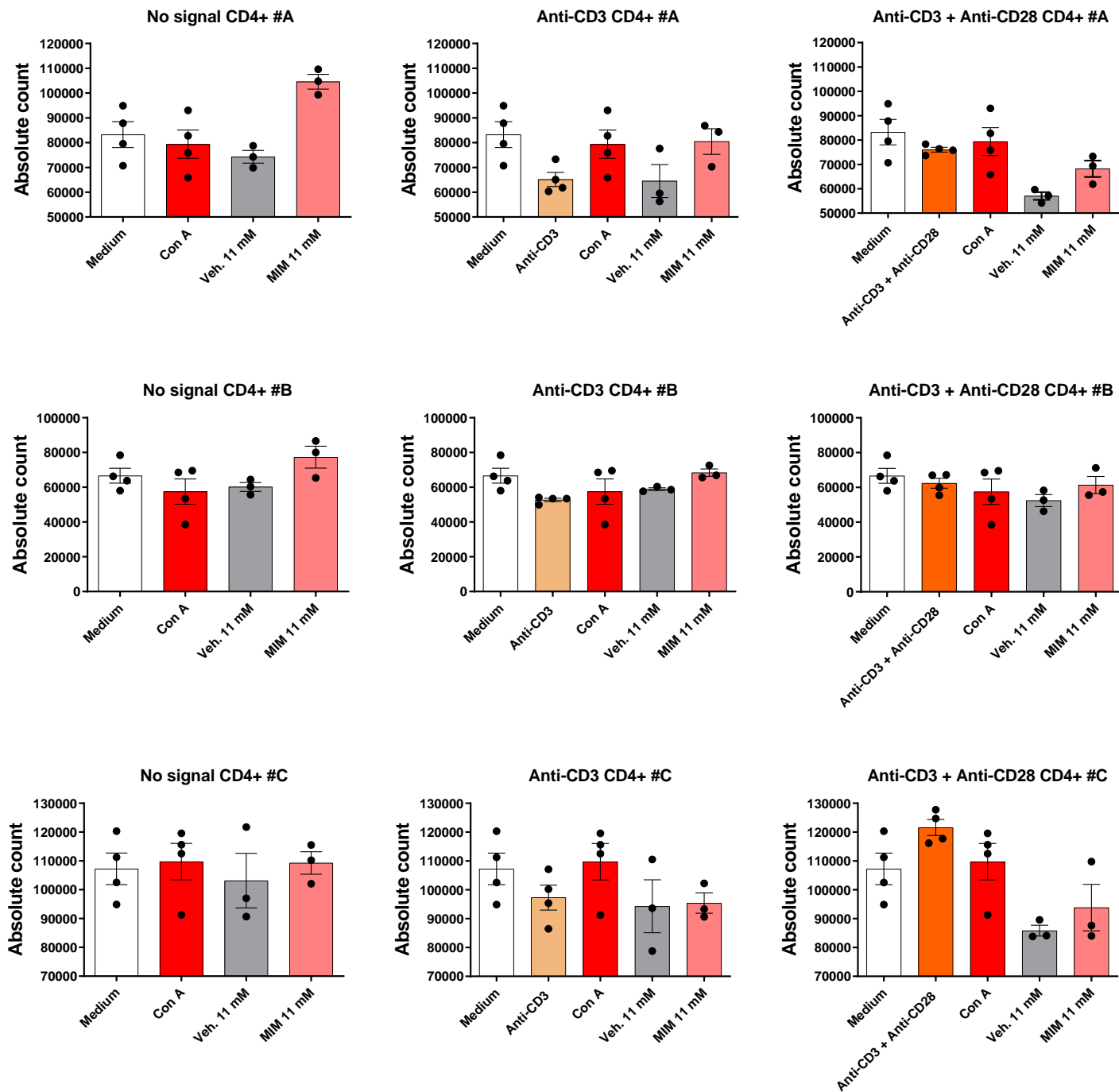

E

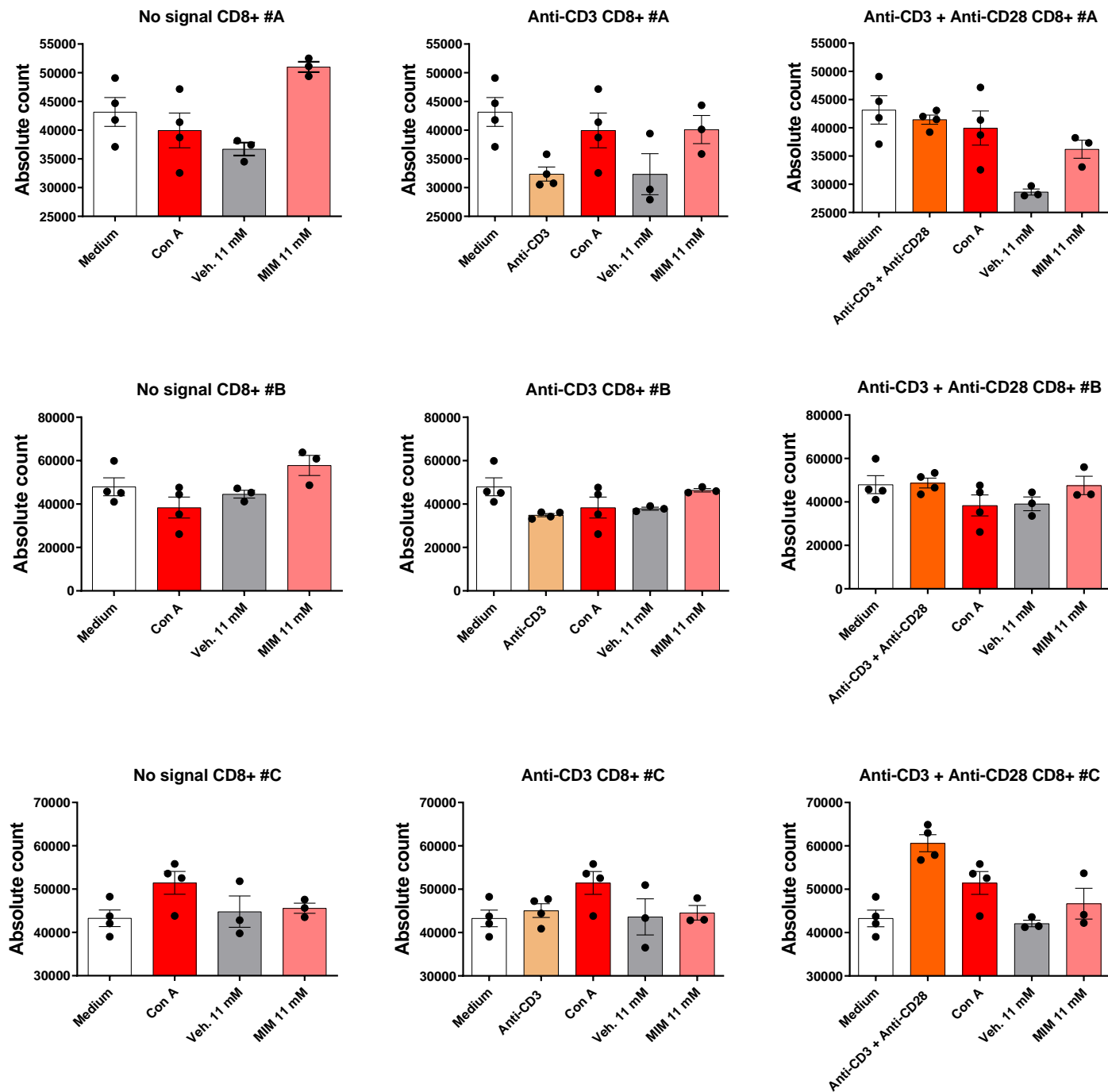

Supplement: Supplementary file 1 [file ijms-23-00110-s001.zip › Sup Figure S2.pdf]

**A**

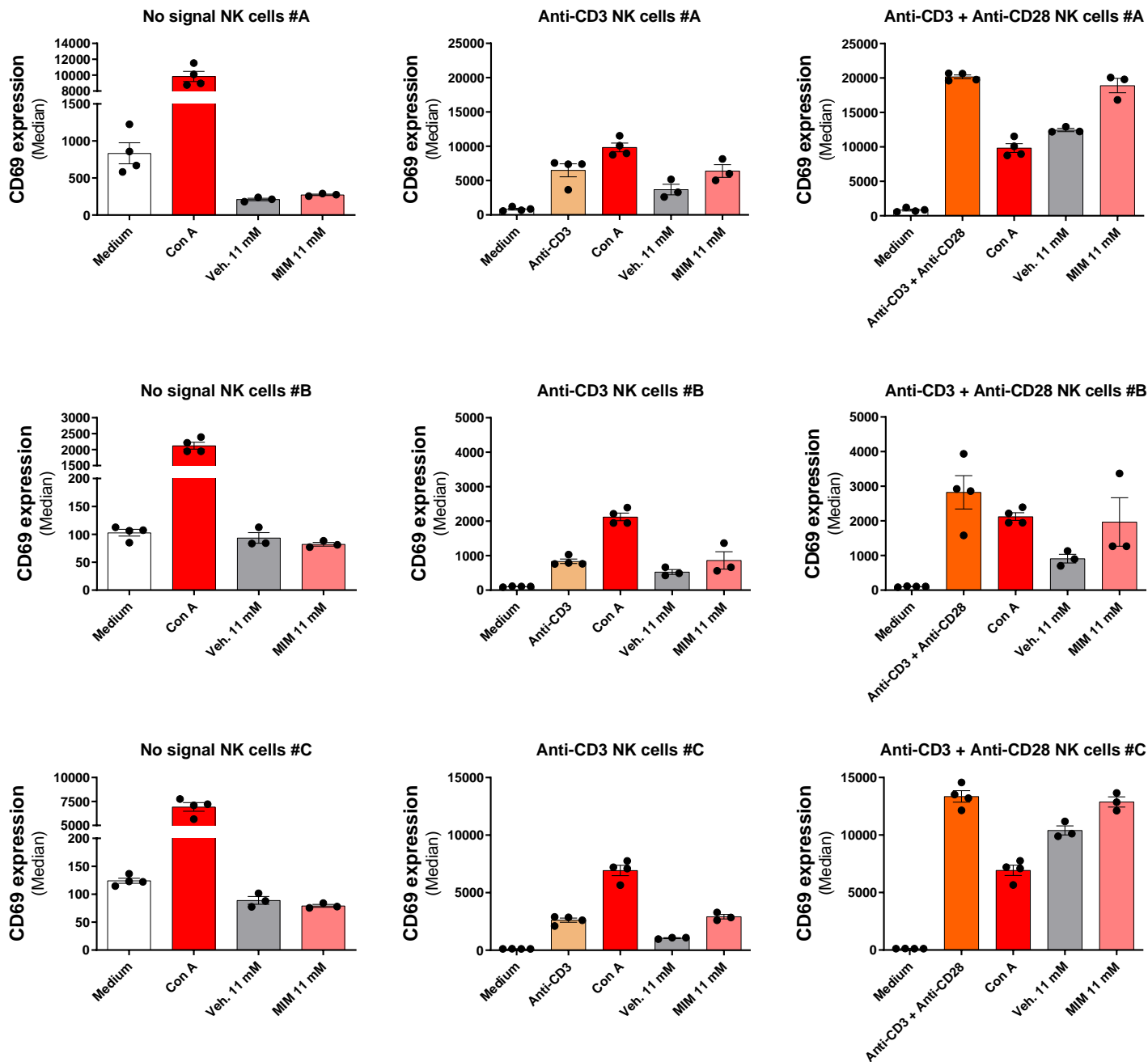

**B**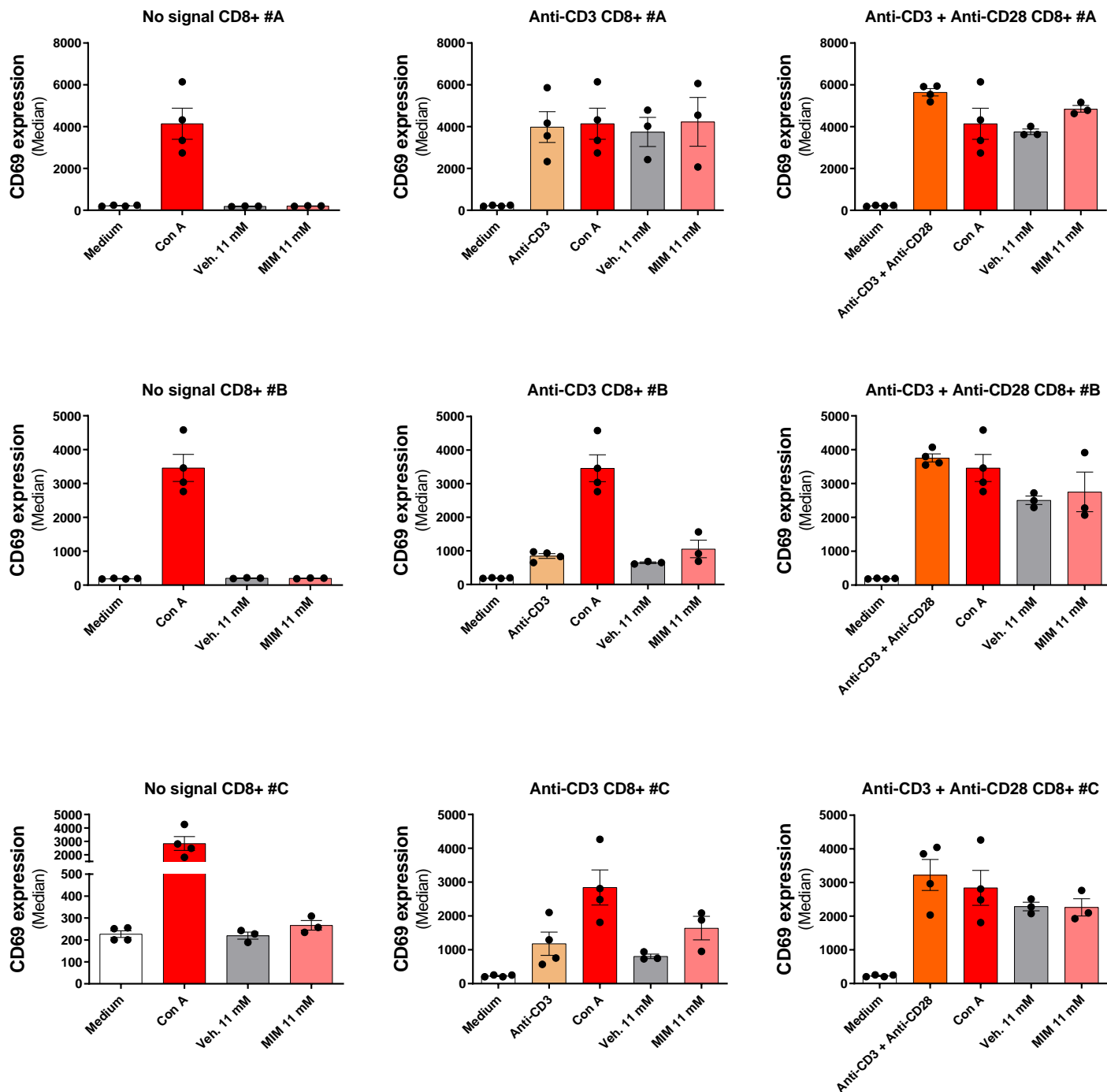

Supplement: Supplementary file 1 [file ijms-23-00110-s001.zip › Sup Figure S3.pdf]
